# A mixed-methods validation of the Intuitive Eating Scale-2 for use with kidney transplant recipients

**DOI:** 10.1371/journal.pone.0340998

**Published:** 2026-01-21

**Authors:** Rebecca J. Linnett, Stephanie J. Hubbard, Noelle Robertson, John Maltby

**Affiliations:** 1 Department of Population Health Sciences, University of Leicester, Leicester, United Kingdom; 2 School of Psychology and Vision Sciences, University of Leicester, Leicester, United Kingdom; Japanese Red Cross Medical Center, JAPAN

## Abstract

Chronic kidney disease (CKD) affects approximately 850 million people worldwide, imposing not only substantial health burdens but also complex dietary management challenges. For kidney transplant recipients (KTRs), dietary restrictions often lessen post-transplant, yet the transition to a ‘normal’ diet is fraught with difficulty after years of externally imposed rules. In this context, intuitive eating, which emphasises internal hunger and satiety cues over external dietary mandates, may support the development of sustainable, self-managed eating behaviours. Despite the recent publication of a third version of the scale, the Intuitive Eating Scale-2 (IES-2) is currently the most widely-used measure of intuitive eating, but it has not yet been validated for use with KTRs. To address this gap, a mixed-methods validation study was conducted, integrating qualitative thinkaloud interviews with KTRs and a quantitative survey involving KTRs and a non-CKD comparison group, to evaluate the face, factorial and construct validity and internal consistency of the IES-2 within this population. Results from both qualitative and quantitative workstreams revealed critical psychometric and conceptual issues with the ‘Unconditional Permission to Eat’ (UPE) subscale, including compromised item discrimination, construct validity, and reliability, as well as responses being systematically influenced by participants’ experiences as KTRs. The original four-factor structure of the IES-2 was unsupported in both groups. Alternative models were tested, and an 11-item, three-factor structure excluding the UPE subscale demonstrated excellent fit across KTR and comparison samples. Although a third version of this scale now exists, there is no validation data available for people with kidney disease or solid organ transplantation, meaning these findings provide the first validated, contextually appropriate configuration of a scale of intuitive eating for use with KTRs, offering a robust tool to advance research and clinical practice in this population.

## 1 Background and rationale

### 1.1 Chronic kidney disease and the ‘kidney diet’

Approximately 850 million people worldwide have chronic kidney disease [1], a condition characterised by a long-term deterioration in kidney functioning [2–4]. Chronic kidney disease can be asymptomatic during its early stages, but as it increases in severity it is often associated with fatigue, weakness, musculoskeletal pain, sleep problems and skin discomfort [5]. Once kidney function drops below 10% (known as end-stage kidney disease), kidney replacement therapy is usually required in the form of haemodialysis, peritoneal dialysis or a kidney transplant [6].

During end-stage kidney disease and dialysis, people with chronic kidney disease are advised to follow a significantly restricted diet and limit fluid intake in order to minimise accumulation of potassium, sodium and phosphorus, and prevent fluid overload [7]. The ‘kidney diet’ has been found to be a considerable burden on people with chronic kidney disease due to the level of restriction it requires [8], with people feeling socially isolated by their diet [9–11] and deprived by what they see as externally-imposed restrictions [12,13]. Furthermore, the dietary guidance is individualised to each person and may change according to blood test results, meaning that it is a difficult diet to understand and maintain [11]. Once a person has received a kidney transplant, however, the strict dietary restrictions they needed to follow prior to transplantation may no longer be necessary. Many people are simply advised to follow a ‘healthy diet’, which could be a considerable challenge for kidney transplant recipients, who may have spent several years on a restrictive diet due to dialysis and/or end-stage kidney disease [14].

### 1.2 Intuitive eating

Within this context, one practice which could be particularly useful for people with chronic kidney disease is that of intuitive eating, also known as ‘normal eating’ or ‘non-dieting’ [15], an approach developed in 1995 by two American dieticians as a result of working with clients who struggled with chronic dieting and weight cycling [16]. Intuitive eating is characterised by four main attributes: 1) Unconditional permission to eat when hungry, without the imposition of external restrictions; 2) Eating for physical rather than emotional reasons; 3) Reliance on internal hunger and satiety cues to determine what, how much and when you eat; and 4) Body-food choice congruence, which focuses on honouring your health and eating food that makes you feel well [17–19]. Whilst there are no studies to date that investigate intuitive eating specifically within the context of kidney transplantation, there is modest evidence to suggest that it is associated with lower levels of disordered eating generally [20,21] and lower cholesterol, BMI and blood pressure [15,22]. It has also been suggested as a potentially useful form of nutritional counselling to improve adherence to dietary recommendations for people with chronic kidney disease [23].

### 1.3 Study rationale

The Intuitive Eating Scale-2 [IES-2]; [18] is a 23-item self-report measure of intuitive eating designed to tap into the four dimensions outlined in the previous section. Although a third iteration of the scale has been developed since the present research took place [24], validation data about the IES-3 outside of the context within which it was developed is still scarce and therefore the IES-2 is still presently the most commonly-used measure of intuitive eating within academic research and clinical practice. Given the subject-matter of the IES-2 and the level of dietary restriction that kidney transplant recipients will have been required to follow pre-transplant, it is reasonable to assume that some items within the scale may not perform the same way in this population as they have done in more general population, non-medical samples. For instance, one of the items in the ‘Unconditional permission to eat’ subscale, “I do not follow eating rules or dieting plans that dictate what, when, and/or how much to eat,” could potentially be answered very differently by a kidney transplant recipient as compared to the college students with whom the scale was originally developed. Whilst many people in this population will no longer need to follow the restrictive kidney diet that they needed to follow pre-transplant, it is possible that the extended period of time that they would have spent with these dietary restrictions may have altered their thoughts, feelings and behaviours surrounding eating, and that they may no longer eat as intuitively as they did prior to developing end-stage kidney disease. No studies to date have evaluated the reliability and validity of the IES-2 within this population and this study therefore aims to address this gap in the literature; whilst intuitive eating could be a useful and beneficial approach for kidney transplant recipients, it is essential that any measures of the construct are appropriate for the unique needs and experiences of this population. Furthermore, this study offers an opportunity to evaluate whether core intuitive eating constructs, particularly the concept of ‘unconditional permission to eat’, are universally applicable across different health contexts. It examines the assumption that intuitive eating behaviours developed in healthy populations can be readily transferred to clinical groups who experience medical dietary restrictions.

## 2 Overall research design

### 2.1 Research question, aims and objectives

This study aimed to assess the suitability of the IES-2 for use with kidney transplant recipients by addressing the following research question:

*To what extent is the Intuitive Eating Scale-2* [18] *a valid and reliable measure of intuitive eating for kidney transplant recipients?*

This was achieved via the following objectives:

1. Evaluate the face validity of the IES-2 using thinkaloud interviews with kidney transplant recipients (Workstream A)
2. Evaluate the factorial and construct validity, and internal consistency reliability, of the IES-2 in this population using a survey of kidney transplant recipients and a comparison group of people without kidney disease (Workstream B)
3. Make comparisons of the reliability and validity of the IES-2 in kidney transplant recipients compared to a comparison group of people without kidney disease (Workstream B)
4. Identify what aspects of the IES-2 (if any) are less relevant for kidney transplant recipients or incompatible with their self-management
5. Make recommendations about how valid the IES-2, in its current form, is for use with kidney transplant recipients

### 2.2 Design

This study incorporated two distinct workstreams: (1) Thinkaloud interviews with kidney transplant recipients; and (2) An online survey for kidney transplant recipients and a comparison group who did not have kidney disease. The first of these workstreams consisted of a series of qualitative interviews and the second consisted of a quantitative survey. Together, these formed a ‘convergent parallel’ research design as defined by Creswell and Plano-Clark’s [25] typologies of mixed methods research. This means that the qualitative and quantitative data were collected and analysed separately during the same phase of the research process and that the results were then mixed during the interpretation phase, using a weaving approach [26], to form an overall impression of the validity of the IES-2. The two workstreams were conducted concurrently to enable a concurrent triangulation design, maximising the rigour and ecological validity of the validation process. This approach allowed immediate comparison between participants’ lived experiences and the psychometric performance of the IES-2, enhancing the trustworthiness and robustness of the findings. Conducting the workstreams in parallel also ensured that emergent issues could be cross-validated in real time, avoided biases that might arise from sequential data collection, and better reflected the complex, multifaceted nature of intuitive eating behaviours among kidney transplant recipients.

Ethical approval for this study was granted on 22/07/2021 by the University of Leicester’s Medicine and Biological Sciences Research Ethics Committee (ref: 26219-rjl48-ls:healthsciences). Informed consent to participate and to publish anonymous and/or aggregate data was received in writing from all participants prior to involvement in the study. The study was conducted in accordance with the Declaration of Helsinki.

## 3 Workstream A: Thinkaloud interviews

Thinkaloud interviews are a form of cognitive interviewing [27] that allow researchers to gain a detailed understanding of what participants are thinking when they are completing a questionnaire [28]. The purpose of conducting these interviews was to assess the face validity of the IES-2 for kidney transplant recipients, and to identify whether there are any limitations to its use in its current form or whether there are any ways in which it may be incompatible with the unique needs and concerns of this population. This workstream is reported in accordance with the Standards for Reporting Qualitative Research (SRQR) checklist [29].

### 3.1 Method

#### 3.1.1 Participants.

Participants were primarily recruited via Twitter. An advertisement was shared on the lead researcher (RL)’s timeline and was re-shared on social media by organisations and charities relating to kidney disease and research. Several kidney disease charities also shared the study on their websites and via email to people signed up to their mailing lists. Based on the sample sizes of similar thinkaloud studies [28,30–32] this study aimed to recruit 6−12 participants as it was felt that this was likely to ensure a broad range of responses to the IES-2. Participants were recruited from the UK only due to the constraints of the study’s ethical approval.

#### 3.1.2 Measures.

**Demographic information.** Once they had given consent, participants were asked to provide their age, gender identity, ethnicity, education level, occupational status and to indicate whether English was their first language. They were also asked to fill in a health questionnaire which asked when they were first diagnosed with chronic kidney disease, details about their current transplant, details about whether they had been on dialysis, and information about any other health conditions they might have.

**Intuitive eating.** Intuitive eating was measured using the 23-item Intuitive Eating Scale-2 [IES-2]; [18]. Items are responded to on a five-point Likert scale which ranges from ‘Strongly Disagree’ to ‘Strongly Agree’. Seven of the items were reverse-scored, and higher scores indicate higher levels of intuitive eating. The IES-2 has four subscales: (1) Unconditional permission to eat; (2) Eating for physical rather than emotional reasons; (3) Reliance on hunger and satiety cues and (4) Body-food choice congruence. The scale includes items such as *“If I am craving a certain food, I allow myself to have it*” and “*I find other ways to cope with stress and anxiety than by eating*”. Participants were asked to complete the IES-2 whilst thinking aloud (see following section) and therefore results relating to this scale were mainly analysed qualitatively in terms of thinkaloud responses rather than in terms of actual quantitative answers to the scale.

#### 3.1.3 Procedure.

Interviews were conducted over the telephone by a research assistant between 01/03/2022 and 31/05/2022. Interviews were digitally recorded on an encrypted device and then transcribed verbatim by a transcriber. Prior to the interviews, people who had shown an interest in participating were sent a link to the online information sheet and consent form. If they decided to participate and completed the consent form, they were then asked to complete the demographic and health questionnaires. Once these questionnaires were complete, participants were given a link to the online survey containing the IES-2; this was password-protected so that it could not be completed before the interview. Participants were assigned a code to link their demographic and health questionnaire data with their IES-2 interview data.

On the day of the interview, the research assistant began the call by giving participants the password to access the online IES-2 questionnaire. They then explained the thinkaloud process using a standardised script adapted from Green and Gilhooly [33]. Participants were then asked to complete a practice exercise to check that they understood the thinkaloud technique. Once this exercise was complete, they were asked to complete the IES-2 in the same manner.

#### 3.1.4 Analysis.

Interview data were analysed using framework analysis [34], which is a widely-used analytic approach in situations where research questions and study objectives are tightly-defined. Transcripts were checked closely by RL, along with the interview reflections provided by the research assistant who conducted the interviews, and participants were then sent copies of the transcripts of their interviews and given the opportunity to retract or clarify anything that they wished to.

The study used a mainly deductive approach to coding, adopting a set of pre-defined codes adapted from an existing framework of response issues from another thinkaloud validation study [28]. The coding scheme was as follows:

1 **No problems** – indicating that participants did not seem to have any problems in understanding and responding to the item
2 **Re-read or stumbled in reading** – suggesting that the participant had difficulty understanding the question
3 **Difficulty generating an answer** – either because the participant had difficulty understanding the question or because it wasn’t applicable to them
4 **Difficulty with the response format** – where participants expressed difficulty in knowing how to answer
5 **Questioned content** – indicating that there may have been problems with how the question was worded
6 **Confusion or misinterpreted** – where participants indicated that they didn’t understand the question, or answered a different question to the one that was being asked

In addition to these codes, a further three inductive codes were generated during initial coding and added to the scheme:

7 **Not relevant to participant’s experience** – where the lack of relevance didn’t create difficulty generating an answer (as in code 4) but may have influenced the answer given
8 **Answer affected by kidney disease** – where the participant’s response appeared to have been influenced by factors related to having chronic kidney disease
9 **Other**

Interviews were coded by RL and then discussed with the research team where necessary. The coder was not formally trained but was immersed in the data and had undertaken extensive research in the process of developing the coding scheme. Once the data had been coded, the number of problem codes per subscale and item were tabulated and then the qualitative responses were analysed, grouped by subscale and then code. Comparisons were made between participants’ responses on each item and the major points that had been made for each subscale. Particular attention was drawn to responses relating to the ‘Answer affected by chronic kidney disease’ code as the primary aim of this study was to assess whether participants’ responses to the IES-2 were affected by them being a kidney transplant recipient.

### 3.2 Results

#### 3.2.1 Participants.

The final sample consisted of nine kidney transplant recipients with an average age of 49.33 years (*SD* = 11.78 years). The sample consisted of five women and four men and was predominantly white (seven participants), with all participants indicating that English was their first language. Participants had been diagnosed with kidney disease for an average of 40 years (*SD* = 21.67 years) and had been transplanted for an average of 7 years (*SD* = 2.56 years). Five (55.6%) had been on dialysis (of any type), for an average of 35.60 months (*SD* = 31.29 months). See Table 1 for full sociodemographic characteristics of the sample.

**Table 1:** Demographic characteristics of thinkaloud sample (N = 9).

*Age (years)*	49.33 (11.78)
Range	32-66
*Gender identity*	
Female	5 (55.6)
Male	4 (44.4)
*Ethnicity*	
Asian/Asian British	1 (11.1)
Mixed/multiple ethnic groups	1 (11.1)
White	7 (77.8)
*Highest education level*	
Postgraduate degree	5 (55.6)
Undergraduate degree	1 (11.1)
Higher education	1 (11.1)
A-level	2 (22.2)
*Occupation*	
Employed part-time	3 (33.3)
Employed full-time	2 (22.2)
Unemployed – looking for work	1 (11.1)
Unable to work	2 (22.2)
Retired	1 (11.1)
*Years since diagnosis*	20.00 (7.50-36.50)^†^
Range	4-40
*Years since current transplant*	2.00 (0.50-4.50)^†^
Range	0-7

Figures denote Mean (*SD*) or *n* (%) unless otherwise stated.

^†^Median (25^th^-75^th^ centile).

#### 3.2.2 Problems identified.

Overall, participants experienced minimal problems with the IES-2. The total number of problems encountered was 72 out of a possible 1,449 (i.e., if all nine participants had flagged issues under every problem code for every item of the IES-2). Problems identified for each subscale are summarised in Table 2 and a mean problem score was calculated for each subscale by dividing the total number of problems for each subscale by the number of items it contains. Items in the ‘Unconditional permission to eat’ and ‘Eating for physical rather than emotional reasons’ subscales accounted equally for the most problems (x̄ = 3.50) and those in the ‘Reliance on hunger and satiety cues’ subscale accounted for the least (x̄ = 2.50). An item-by-item tally of problems identified is available in the S1 Table which also shows that the subscale most affected by the respondent having chronic kidney disease was ‘Unconditional permission to eat’ (nine problems across six items) and the subscale least affected was ‘Eating for physical rather than emotional reasons’ (five problems across eight items).

**Table 2:** Total problems across IES-2 subscales.

	Labels									Mean # of problems^a^
	No problems	Re-read or stumbled reading	Difficulty generating an answer	Difficulty with response format	Questioned content	Confusion or mis-interpreted	Not relevant to participant	Answer affected by CKD	Other	
UPE	37	1	4	0	7	0	0	9	0	3.50
EPR	48	3	3	3	7	1	6	5	0	3.50
RHSC	41	1	0	0	6	0	0	8	0	2.50
BFCC	20	0	0	1	3	0	0	3	1	2.67

^a^Total number of problems for subscale divided by number of items in subscale.

UPE = *Unconditional permission to eat* subscale (6 items).

EPR = *Eating for physical rather than emotional reasons* subscale (8 items).

RHSC = *Reliance on hunger and satiety cues* subscale (6 items), BFCC = *Body-food choice congruence* subscale (3 items).

**‘Unconditional permission to eat’ subscale.** The items in this subscale are focused on the idea that no food is inherently ‘bad’ or ‘forbidden’ and that people instead have unconditional permission to eat what and when they want. Overall, this subscale had the greatest number of instances of participants’ responses being affected by having chronic kidney disease.

The item that yielded the most problems was *“I have forbidden foods that I don’t allow myself to eat”*, with four out of nine participants giving answers that indicated that their response was affected by them living with chronic kidney disease. This was mostly focused around food restrictions related to anti-rejection medication but also uncooked or otherwise risky foods such as sushi which they avoided due to being immunocompromised. Participants that raised this as an issue tended to agree or strongly agree with this item, indicating lower levels of intuitive eating because this item is reverse-scored:

*Well, only on medical grounds... which was grapefruit and red wine... So I have forbidden foods. So, I strongly agree. “I have forbidden foods that I don’t allow myself to eat.”.. Strongly agree.* (TN, male, age 59)

Despite this, answers to this item overall were skewed towards ‘Strongly disagree’ and ‘Disagree’, indicating higher levels of intuitive eating. In fact, some participants indicated that they had to be on such a restricted diet pre-transplant that this has meant that they don’t impose any restrictions on themselves now that it is not medically necessary:

*I used to, but certainly not any more. There is nothing that I wouldn’t have something of because I went without it for so long. So I would say, no... there’s nothing forbidden any more.*

(AF, female, age 40)

Another item in this subscale that yielded several problems was, “*I do not follow eating rules or dieting plans that dictate what, when and/or how much to eat*”. Given that the IES-2 was developed in a sample of college students, this item is likely referring to weight-loss diets or the exclusion/restriction of certain macro-nutrients, but many of the participants in this study interpreted it in relation to their experience of being on a restricted diet prior to transplantation or in terms of kidney-specific measures they needed to take now to ensure the longevity of their transplant. This seemed to pose difficulties for several participants in knowing how to answer, which may account for why there was a large proportion of ‘Neither agree nor disagree’ answers to this question (44.4%). Some participants expressed that they don’t necessarily self-impose restrictions but that there are certain ‘rules’ that they *have* to follow, particularly with regards to timing of eating around medication and also the medication-related food restrictions already discussed:

*I have to follow certain rules around my medication... I’m gonna do a very middling response because there are rules that I* have *to follow...*

(CT, male, age 32)

The other issue raised around this item was that it could be considered to ask several questions in one (i.e., ‘what’, ‘when’ and ‘how much’) which is generally to be avoided during item construction [35] and which led to some participants expressing confusion about how to interpret and answer the question.

The same issue was raised with another item in this subscale, “*I try to avoid certain foods high in fat, carbohydrates or calories*”:

*There are three questions here in fact, so this should be three types of questions, because they’re different... I’d put it in separate questions, and I think you have to qualify what you mean... Calories is obvious, but carbohydrates can mean different things...*

(LG, female, age 66)

As highlighted above, this question also doesn’t differentiate between different types of fats or carbohydrates, which led to difficulty answering for some participants:

*It depends what you mean by fat... Because I eat high in fats... but it’s the good fat...*

(FG, female, age 62)

The mean score for this subscale among the thinkaloud participants was 3.33 (*SD* = 0.51) which is within the expected range for a scale with 5 response points.

**‘Eating for physical rather than emotional reasons’ subscale.** The items in this subscale are focused on eating due to physical hunger rather than as a source of emotional support or comfort. Overall, this subscale had the fewest instances of responses being affected by chronic kidney disease, but there were still other issues raised, particularly around the wording and content of some of the questions.

One issue that came up with two of the items in this subscale is that they refer to loneliness (“*I find myself eating when I am lonely, even when I’m not physically hungry*” and “*When I am lonely, I do not turn to food for comfort”*). Interestingly, these questions posed difficulties for some participants as they didn’t feel that they were ever lonely and therefore didn’t know how to answer the question:

*Because being alone, is not being lonely. Being lonely is having nothing, nobody... And I never actually, I’ve never actually been like that... So... I don’t know whether I can answer that question other than a 3, I think... You could do with a ‘Not available’.*

(FG, female, age 62)

However, for other participants, the opposite was true, and the theme of loneliness was inextricably linked to their identity as a kidney transplant recipient due to their need to shield from COVID-19:

*I have done a lot of that [eating when lonely] over the last two years... it’s like I’m feeding that emotional gap... that I would normally get from seeing friends, hugging friends, going to my work... Just living my life pre-COVID, basically.*

(ML, female, age 50)

For these participants, answers to these questions about loneliness as well as answers to the question “*I find myself eating when I’m feeling emotional (e.g., anxious, depressed, sad), even when I’m not physically hungry”* were not only affected by the respondent being a kidney transplant recipient but by them being a kidney transplant recipient in this specific socio-historical context:

*I guess this pandemic has made us be lonely to a certain extent... A lot of immune compromised people have been shielding.*

(CB, male, age 36)

The mean score for this subscale among the thinkaloud participants was 3.63 (*SD* = 0.96) which is within the expected range for a scale with 5 response points.

**‘Reliance on hunger and satiety cues’ subscale.** The items in this subscale are focused on eating in response to internal hunger and fullness cues rather than relying on external cues such as calorie or macronutrient counting. Overall, this subscale had the second-greatest instances of responses being affected by chronic kidney disease.

One of the most frequent issues raised with the items in this subscale was the difficulty with relying on internal hunger and satiety cues when taking anti-rejection medications, due to the increased appetite associated with many of these. For example, in response to the items, “*I rely on my fullness (satiety) signals to tell me when to stop eating*” and “*I trust my body to tell me how much to eat*”, several participants indicated that they were unable to do this and that the decision to stop eating has to be made intellectually rather than as a result of listening to their body:

*I guess the person who wrote this has never been on Pred [Prednisolone]... When you are on Pred there is no such thing as being full, you could just keep eating...*

(CB, male, age 36)

The mean score for this subscale among the thinkaloud participants was 3.26 (*SD* = 0.76) which is within the expected range for a scale with 5 response points.

**‘Body-food choice congruence’ subscale.** The items in this final subscale are focused on the idea of ‘gentle nutrition’ and choosing foods that give you energy and make you feel well. There are only three items in this subscale and one yielded no problems at all; with the other two (“*I mostly eat foods that make my body perform efficiently (well)*” and “*I mostly eat foods that give my body energy and stamina*”), some participants interpreted the ideas of ‘performing efficiently’ and eating for stamina as something that was not relevant to their lives:

*When I see that phrase, ‘perform efficiently’, it makes me think of an athlete... So I, I just need my body to do certain things, I don’t need to be able to run 100 metres in 8 seconds...*

(AG, female, aged 52)

One participant also said that their focus was on keeping their transplant stable and healthy, rather than on efficiency or stamina:

*My main concern generally isn’t making my body perform efficiently... not my body as a whole, anyway... I am more concerned about my kidney function... I need to make sure this transplant works.*

(CB, male, age 36)

The mean score for this subscale among the thinkaloud participants was 3.74 (*SD* = 1.14) which is within the expected range for a scale with 5 response points.

### 3.3 Discussion

The aim of Workstream A was to evaluate the face validity of the IES-2 for kidney transplant recipients, in order to contribute to the overall aim of the validation study of assessing whether the IES-2 is a valid and reliable measure of intuitive eating in this population. Considering the length of the scale and the number of potential problem codes that could have been identified, the participants in this workstream encountered minimal problems.

One of the main problems that this workstream identified that was specifically related to the respondent being a kidney transplant recipient was that many participants responded to questions about ‘forbidden’ foods and eating plans in terms of risky foods they have to avoid due to immunocompromisation, medication-incompatible foods and ‘rules’ to do with timing of eating around medication. These questions were all part of the ‘Unconditional permission to eat’ subscale, which also had the most instances of responses being affected by chronic kidney disease. This observation suggests that the intuitive eating framework may contain implicit assumptions about the availability of unrestricted food choices — assumptions that may not hold in populations subject to medical dietary restrictions. Consequently, intuitive eating models may require reconceptualisation when applied to groups with chronic illness to ensure theoretical and practical relevance. This is similar to the findings of Paterson et al.’s [31] study validating the original Intuitive Eating Scale [17] in a sample of pregnant women, which found that food safety issues relating to pregnancy changed participants’ responses to certain questions. They suggested that adapted instructions could be provided to clarify what these questions are really asking, which could also be useful for kidney transplant recipients. For example, instructions could clarify whether questions about ‘forbidden foods’ are referring to foods that they avoid due to infection risk or medication incompatibility, or whether such questions are talking more generally about foods that they avoid perhaps because they consider them ‘addictive’ or ‘unhealthy’ (which is likely to be closer to what the question was really trying to gauge). Interestingly, this is something that the newest iteration of the Intuitive Eating Scale [IES-3]; [24], has sought to address, as it contains instructions at the beginning that state “Specifically, we are interested in your approach to eating foods that are available to you: meaning, foods that you have access to, can afford, and don’t have a medical or value-based reason for avoiding” (p. 16).

Most of the other problems identified within this workstream were not specific to this population but were about the scale more broadly. For example, there were several questions that were ambiguously worded, such as *“I try to avoid certain foods high in fat, carbohydrates, or calories”*, which participants struggled to answer because the question didn’t make a distinction between (for example) ‘good’ and ‘bad’ fats. This can be problematic as it does not make it clear what the question is or is not referring to, which can result in participants guessing at the meaning or choosing middle options as a means of ‘opting out’ of the question or expressing that they don’t know [36,37].

#### 3.3.1 Strengths and limitations.

A strength of the thinkaloud method used for this workstream is that problems flagged with questionnaire items in thinkaloud studies have been found to often point to problems in responding (such as an increased likelihood of endorsing the middle option on a five- or seven-point Likert scale), which can lead to invalid measurements and an increase in measurement error [36,38]. This means that, through qualitative insights, the thinkaloud method can often highlight areas where there may be quantitative problems with an instrument. However, it is dependent on the participant’s ability to self-reflect on their thoughts, feelings and behaviours and then to be able to verbalise what they’re thinking, and consideration also needs to be given about the coding scheme used, in terms of whether the observable behaviour reflected in the codes actually means what the coding scheme infers it does.

The thinkaloud method does not usually allow participants to elaborate on their responses; instead, the interviewer remains silent unless they are prompting the participant to continue to speak after a period of silence. However, as Aujla et al. [28] point out, this may not be the most ethical approach in clinical populations, as it restricts participants from being able to tell their story. Instead, Beatty and Willis [39] suggest asking specific, direct questions about how participants have come to the response they have given, which is the approach that was taken in this workstream. This is a strength of the current study, as participants were given the opportunity make their voices heard and the interviewer was able to dig deeper into the issues that participants felt needed to be addressed with questions they found problematic. This also helped to achieve data saturation, as by gaining more detailed responses from participants it was possible to see that a broad range of perspectives were represented, with participants confirming the experiences and views of each other as well as presenting opposing views at times. By the end of the interviews, it was felt that no new insights were being provided and that data saturation had therefore been achieved.

## 4 Workstream B: Survey

### 4.1 Method

#### 4.1.2 Participants.

Based on recommendations by Nunnally and Bernstein [40] and Velicer and Fava [41], this study aimed to recruit 230 participants for each group (i.e., 10 participants per item on the IES-2). Participants were recruited online; advertisements were shared on the lead author’s social media feeds and were also shared on social media by organisations and charities relating to health psychology or kidney research. Participants were also recruited (following admin approval) via advertisements on Facebook groups for people with kidney disease and/or a solid organ transplant, as well as emails to several academic mailing lists. Several kidney disease charities also shared the study on their websites and via email to people signed up to their mailing lists. The survey ran from 01/07/2021 to 31/08/2022. Participants were recruited from the UK only due to the constraints of the study’s ethical approval.

#### 4.1.3 Measures.

**Demographic information.** Once they had given consent, participants were asked to provide demographic information as outlined in Section 3.1.2.

**Intuitive eating.** Intuitive eating was measured using the 23-item Intuitive Eating Scale-2 [IES-2]; [18], as outlined in Section 3.1.2. In the original development and validation study of the IES-2 [18], internal consistency (α) was good amongst women and men, respectively, for the IES-2 total score (.85/.88) and for each of the four subscales: ‘Unconditional permission to eat’ (.77/.82), ‘Eating for physical rather than emotional reasons’ (.92/.92), ‘Reliance on hunger and satiety cues’ (.85/.87) and ‘Body-food choice congruence’ (.87/.84).

**Anxiety.** Anxiety was measured using the 7-item Generalized Anxiety Disorder-7 [GAD-7]; [42]. Items are responded to on a four-point Likert scale ranging from ‘Not at all’ to ‘Nearly every day’ according to how often the respondent has been bothered by the problem described in the last two weeks. None of the items are reverse-scored, and higher scores indicate higher levels of anxiety. The scale includes items such as “Feeling nervous, anxious or on edge” and “Becoming easily annoyed or irritable”. A recent review has found that the GAD-7 has demonstrated good internal consistency (α = .75−.91) across a number of samples [43].

**Body appreciation.** Body appreciation was measured using the 13-item Body Appreciation Scale [BAS; [44]. Items are responded to on a five-point Likert scale ranging from ‘Never’ to ‘Always’ depending on how often the respondent has behaved in the stated manner. None of the items are reverse-scored, and higher scores indicate higher levels of body appreciation. The scale includes items such as “Despite its flaws, I accept my body for what it is” and “I engage in healthy behaviours to take care of my body”. In the original development and validation study of the BAS, internal consistency (α) across the three studies was good (.91−.94). Feedback from a patient and public involvement exercise during the development of this study highlighted that Q.12 of the BAS is gendered (“I do not allow unrealistically thin images of women presented in the media to affect my attitudes toward my body”). This item was reworded in the final survey to “I do not allow unrealistic images presented in the media to affect my attitudes toward my body”.

**Social desirability.** Social desirability was measured using the 5-item Socially Desirable Response Set-5 [SDRS-5; [45]. Items are responded to on a five-point Likert scale ranging from ‘Definitely true’ to ‘Definitely false’, depending on how much the respondent feels that the item is reflective of their relationship with others. Two of the items are reverse-scored, and higher scores indicate higher levels of socially desirable responding. The scale includes items such as “I am always courteous even to people who are disagreeable” and “I sometimes feel resentful when I don’t get my way”. The SDRS-5 has showed adequate internal consistency (α = .76) in recent studies [46].

#### 4.1.4 Analysis.

**Item analysis.** The first stage of the analysis focused on the IES-2 items. Item facility indices were inspected and any items with a frequency index outside the 2–4 range were flagged as being potentially problematic due to having a heavily skewed distribution [47]. Next, any items that had two or more adjacent scale points representing less than 20% of the total responses were also flagged as potentially having problems with frequency distribution. Item-total correlations were used to assess item discrimination, and inter-item correlations (between items within each of the four subscales) were used to assess item validity. Items were flagged as being potentially problematic if they had item-total correlations <0.3 [48], or if more than 50% of their inter-item correlations were either below 0.3 or above 0.8. Spearman’s Rho correlations were used throughout the study due to the ordinal nature of the data produced by Likert scales [35].

**Validity.** Construct validity was then explored. Firstly, convergent and divergent construct validity was evaluated by correlating IES-2 total and subscale scores with measures representing constructs that would be expected to be related to intuitive eating, [e.g. 35,49–51]. Anxiety and body appreciation are known to be negative and positively related to intuitive eating, respectively [18,52] so they were used to test convergent construct validity. Social desirability is known to be unrelated (or negligibly related) to intuitive eating [17,18] so this was used to test discriminant construct validity. Finally, known-groups construct validity was examined. This is based on the principle that certain specified groups of participants might be expected to score differently from others, and that the measure should be sensitive enough to detect these differences [53]. Men have generally been found to have higher levels of intuitive eating than women [e.g. 18] and therefore the known-groups validity of the IES-2 was assessed by performing an independent (2-sample) *t*-test on the data from each sample to test for differences in mean IES-2 total and subscale scores between men and women.

**Reliability. I**nternal consistency reliability was then calculated for the total IES-2 and for each of the subscales using Cronbach’s alpha (α).

**Confirmatory factor analyses.** Four structural models of the IES-2 were then tested as outlined below. These consisted of the original model posited by the IES-2 authors, and several models identified in the literature. The models were specified and analysed in SPSS AMOS v.28, and were estimated using maximum likelihood estimation. The authors of the original development and validation paper [18] recommend correlating the error terms of items that share method effects, such as those that are similarly phrased [see also [54,55]. These were four items that begin “*I trust my body to tell me...*” (items 6, 7, 8 and 23), three items that begin “*I find myself eating when I am...*” (items 2, 5 and 11), two items that begin “*I mostly eat foods that...*” (items 19 and 20) and two items that end *“... turn(ing) to food for comfort”* (items 12 and 14). Each of the following models were run with and without correlated errors in order to assess which approach provided the best fit.

1 **Model 1: Original higher-order four-factor model.** Hierarchical model with 23 items loading onto four latent variables representing the four subscales of the instrument – (1) Eating for physical rather than emotional reasons, (2) Unconditional permission to eat, (3) Reliance on hunger and satiety cues, and (4) Body-food choice congruence – which then load onto a higher-order latent variable of total intuitive eating. This is the factor structure set out in the original development and validation paper of the IES-2 [18].
2 **Model 2: Correlated four-factor model.** 23 items loading onto four latent variables representing the IES-2 subscales, with the latent variables inter-correlated. This factor structure has been posited by a number of studies [56–60].
3 **Model 3: Six-factor model.** An alternative model of the IES-2 with 23 items loading onto six latent variables representing the six factors posited by the authors [59]. These factors were (1) Avoiding forbidden foods (items 1, 9 and 4); (2) Permission to eat (items 3, 16 and 17); (3) Avoiding emotional eating (items 2, 5, 10 and 11); (4) Avoiding food-related coping strategies (items 12, 13, 14 and 15); (5) Reliance on hunger and satiety cues (items 6, 7, 8, 21, 22 and 23) and (6) Body-food choice congruence (items 18, 19 and 20).
4 **Model 4: Three-factor short form model.** An alternative model of the IES-2 with 11 items loading onto three latent variables representing the three factors posited by the authors [60]. These factors were (1) Eating for physical rather than emotional reasons (items 2, 5, 10 and 11); (2) Reliance on hunger and satiety cues (items 6, 7, 8 and 23) and (3) Body-food choice congruence (items 18, 19 and 20).

To evaluate the model fit using confirmatory factor analysis, we employed standard goodness-of-fit indices as recommended by Hu and Bentler [61] and Kline [55]. These included the relative chi-square (CMIN/DF), alongside the chi-square and degrees of freedom, the comparative fit index (CFI), the non-normed fit index (NNFI), the root mean square error of approximation (RMSEA), and the standardised root mean square residual (SRMR). Model fit was assessed through two complementary approaches. First, we examined whether the fit indices met conventional thresholds, defined as a CMIN/DF less than 3, CFI and NNFI values greater than 0.90, and RMSEA and SRMR values below 0.08 [61–63]. Second, we evaluated the incremental validity of the proposed models, following the approach outlined by Barrett [64]. Improvements in model fit were assessed by examining changes in the Comparative Fit Index (CFI), with an increase greater than .01 indicating a meaningful improvement in model fit [65].

### 4.2 Results

#### 4.2.1 Participants.

The final sample consisted of 487 adults (245 kidney transplant recipients and 242 people without kidney disease) living in the UK. The average age was 52.06 years (*SD* = 12.84 years) for the kidney transplant recipient group and 39.98 years (*SD* = 13.25 years) for the comparison group. Both samples were predominantly female, although this was to a greater extent in the comparison group (59.2% of kidney transplant recipients and 88.4% of the comparison group). Both samples were also predominantly white (93.5% of kidney transplant recipients and 86.4% of the comparison group), and the majority of participants spoke English as their first language (96.7% of kidney transplant recipients and 89.7% of the comparison group). Within the kidney transplant recipient group, participants had been diagnosed with kidney disease for an average of 26.40 years (*SD* = 13.46 years) and had been transplanted for an average of 9.66 years (*SD* = 7.97 years). See Table 3 for full sociodemographic characteristics of both samples. All participants (N = 487) completed the 48-item questionnaire. There were no missing data due to the design of the questionnaire on the online platform.

**Table 3:** Demographic characteristics of the survey samples (N = 487).

	KTRs (*n* = 245)	Comparison (*n* = 242)
*Age (years)*	52.06 (12.84)	38.98 (13.25)
Range	20-78	18-82
*Gender identity*		
Female	145 (59.2)	214 (88.4)
Male	98 (40.0)	25 (10.3)
Non-binary/third gender	2 (0.8)	3 (1.2)
*Ethnicity*		
Asian/Asian British	5 (2.0)	12 (5.0)
Black/African/Caribbean/Black British	7 (2.9)	9 (3.7)
Mixed/multiple ethnic groups	3 (1.2)	4 (1.7)
White	229 (93.5)	209 (86.4)
*Highest education level*		
Postgraduate degree	54 (22.0)	149 (61.6)
Undergraduate degree	74 (30.2)	53 (21.9)
Higher education	46 (18.8)	7 (2.9)
A-level	25 (10.2)	18 (7.4)
GCSE or equivalent	37 (15.1)	8 (7.4)
No qualification	4 (1.6)	2 (0.8)
Other, or prefer not to say	5 (2.0)	5 (2.1)
*Occupation*		
Employed part-time	32 (13.1)	47 (19.4)
Employed full-time	74 (30.2)	108 (44.6)
Self-employed	14 (5.7)	13 (5.4)
Full-time student	2 (0.8)	38 (15.7)
Unemployed – looking for work	8 (3.3)	7 (2.9)
Unemployed – not looking for work	7 (2.9)	1 (0.4)
Unable to work	28 (11.4)	9 (3.7)
Retired	71 (29.0)	11 (4.5)
Other, or prefer not to say	9 (3.7)	8 (3.3)
*Years since diagnosis*	26.00 (16.00-37.00)^†^	-
Range	2-65	-
*Years since current transplant*	7.00 (4.00-14.00)^†^	-
Range	1-43	-

KTRs = Kidney transplant recipients.

Figures denote Mean (*SD*) or *n* (%) unless otherwise stated.

^†^Median (25^th^-75^th^ centile).

#### 4.2.2 Item analysis.

Responses for all items on the IES-2 covered the full range of response options (1–5) and all item facility indices were within the acceptable range. Examination of item-total correlations suggested item discrimination problems with four of the six items in the ‘Unconditional permission to eat’ subscale in both the kidney transplant recipient and comparison samples. All other item-total correlations were within acceptable range; see the S2 Table for full item analysis results. Five items were found to potentially have response distribution problems as two or more of their adjacent scale points represented less than 20% of responses; three of these items were from the ‘Unconditional permission to eat’ subscale and one was from each of the ‘Eating for physical rather than emotional reasons’ and ‘Body-food choice congruence’ subscales. Finally, inspection of within-factor inter-item correlations found problems with item validity for all of the items in the ‘Unconditional permission to eat’ subscale for the kidney transplant recipient sample, in that they were weakly correlated (<0.3) more than 50% of the time. See Table 4 for a summary of item analysis problems for each item where problems were identified.

**Table 4:** IES-2 item analysis problems.

		Freq. of response endorsement		Item-total r		Inter-item correlations	
Item	Subscale	KTRs	Comparison	KTRs	Comparison	KTRs	Comparison
IES01	UPE			✗	✗	✗	
IES03	UPE	✗	✗	✗	✗	✗	
IES04	UPE					✗	
IES09	UPE		✗	✗	✗	✗	
IES15	EPR		✗				
IES16	UPE		✗	✗	✗	✗	
IES17	UPE					✗	
IES18	BFCC	✗					

UPE = *Unconditional permission to eat*, EPR = *Eating for physical rather than emotional reasons,* RHSC = *Reliance on hunger and satiety cues*, BFCC = *Body-food choice congruence.*

#### 4.2.3 Validity and reliability.

To test the validity of the IES-2, Spearman’s Rho correlations were performed between the IES-2 and its subscales and anxiety, body appreciation and social desirability.

**Convergent construct validity.** For kidney transplant recipients, total intuitive eating scores and all subscales apart from ‘Unconditional permission to eat’ were significantly positively associated with body appreciation, supporting convergent construct validity. Similarly, for the comparison group, total intuitive eating and all subscales were significantly positively associated with body appreciation. In the case of anxiety, total intuitive eating scores and all subscales were significantly negatively associated with GAD-7 scores in the sample of kidney transplant recipients, supporting convergent construct validity. For the comparison group, total intuitive eating scores and the ‘Eating for physical rather than emotional reasons’ and ‘Body-food choice congruence’ subscales were negatively associated with anxiety but the ‘Unconditional permission to eat’ and ‘Reliance on hunger and satiety cues’ subscales were not.

**Discriminant construct validity.** Social desirability was not significantly associated with total intuitive eating scores nor any of the subscales in either the kidney transplant recipient or comparison group, supporting discriminant construct validity. However, it should be noted that the internal consistency of the scale used to measure social desirability was questionable, but not necessarily poor [66], for both the kidney transplant recipient group (α = .60) and the comparison group (α = .66).

**Known-groups construct validity.** With the exception of the ‘Unconditional permission to eat’ subscale in the comparison group sample, means showed men to have higher levels of total intuitive eating and all subscales. However, a series of independent samples *t*-tests showed that very few of these differences between groups were statistically significant. There were statistically significant differences between male and female means for the ‘Eating for physical rather than emotional reasons’ subscale in both the kidney transplant recipient (female x̄ = 3.29, *SD* = 2.5, male x̄ = 3.58, *SD* = 1.05, *t*(241) = −2.09, *p* = .04, *d* = −.27 (95% CI [−.53, −.02]) and comparison (female x̄ = 2.94, *SD* = 0.97, male x̄ = 3.40, *SD* = 0.82, *t*(237) = −2.28, *p* = .02, *d* = −.48 (95% CI [−.90, −.06]) groups, but otherwise known-groups construct validity was largely unsupported. It should be noted that the samples included several non-binary/third gender participants (kidney transplant recipient group *n* = 2, comparison group *n* = 3) that could not be included in these analyses and therefore there were five fewer participants overall for the purpose of these tests.

**Internal consistency** Cronbach’s alpha for the total IES-2 and all subscales apart from ‘Unconditional permission to eat’ was 0.85 or above in the kidney transplant recipient group and 0.88 or above in the comparison group. Internal consistency for the ‘Unconditional permission to eat’ subscale was inadequate by Carmines and Zeller’s [67] threshold of 0.8 in both the kidney transplant recipient group (α = 0.52) and the comparison group (α = 0.74).

Table 5 contains all correlation and alpha estimates from this study for all scales and subscales used and Table 6 contains results from t-tests used to assess known-groups construct validity.

**Table 5:** Variable correlations, means (*M*), standard deviations (*SD*) and internal consistencies (α).

Variable		1	2	3	4	5	6	7	8	α _KTR_	*M*_KTR_	*SD*_KTR_	Range _KTR_
1.	Total intuitive eating	-	.43***	.83***	.77***	.54***	-.34***	.55***	.09	0.87	3.27	0.66	1-5
2.	UPE	.47***	-	.08	.31***	-.05	-.14*	.12	.01	0.52	3.28	0.72	1-5
3.	EPR	.80***	.09	-	.43***	.45***	-.34***	.52***	.10	0.90	3.41	1.07	1-5
4.	RHSC	.80***	.36***	.43***	-	.31***	-.15*	.31***	.06	0.85	3.03	0.98	1-5
5.	BFCC	.51***	-.08	.35***	.35***	-	-.32***	.51***	.10	0.87	3.37	1.01	1-5
6.	Anxiety	-.22***	-.04	-.25***	-.12	-.15*	-	-.48***	-.15	0.91	1.89	0.75	1-4
7.	Body appreciation	.67***	.29***	.52***	.49***	.47***	-.38***	-	.13	0.95	3.31	0.98	1-5
8.	Social desirability	-.01	-.05	-.05	.06	.06	-.11	.01	-	0.60	3.97	0.63	2-5
	α _Comparison_	0.88	0.74	0.91	0.88	0.90	.91	.95	.66				
	*M*_Comparison_	3.17	3.47	3.00	3.05	3.28	1.95	3.27	3.70				
	*SD*_Comparison_	0.64	0.78	0.97	0.94	1.05	0.73	0.87	0.67				
	Range _Comparison_	1-5	1-5	1-5	1-5	1-5	1-4	1-5	2-5				

Values for the kidney transplant recipient (KTR) group are presented above the diagonal; values for the comparison group are presented below.

UPE = *Unconditional permission to eat* subscale, EPR = *Eating for physical rather than emotional reasons* subscale.

RHSC = *Reliance on hunger and satiety cues* subscale, BFCC = *Body-food choice congruence* subscale.

* *p* < .05 *** *p* < .001.

**Table 6:** Independent samples *t*-tests for differences in intuitive eating scores (*N* = 487).

										Cohen’s *d*		
	Women			Men							95% CI	
	*n*	*M*	*SD*	*n*	*M*	*SD*	*df*	*t*	*p*	Point est.	Lower	Upper
**Kidney transplant recipients**												
Total IES-2	145	3.20	0.70	98	3.36	0.60	241	-1.84	.07	-.24	-.50	.02
UPEa	145	3.24	0.76	98	3.35	0.65	229.1	-1.21	.23	-.15	-.41	-.10
EPR	145	3.29	1.08	98	3.58	1.05	241	-2.09	.04*	-.27	-.53	-.02
RHSC	145	3.00	1.01	98	3.06	0.94	241	-0.51	.61	-.07	-.32	.19
BFCC	145	3.32	1.09	98	3.41	0.88	241	-0.74	.46	-.10	-.35	.16
**Comparison group**												
Total IES-2^a^	214	3.15	0.66	25	3.30	0.43	38.95	-1.52	.14	-.23	-.64	.19
UPE	214	3.50	0.76	25	3.17	0.85	237	2.03	.05	.43	.01	.84
EPR	214	2.94	0.97	25	3.40	0.82	237	-2.28	.02*	-.48	-.90	-.06
RHSC	214	3.03	0.96	25	3.19	0.79	237	-0.77	.44	-.16	-.58	.25
BFCC	214	3.26	1.03	25	3.51	1.14	237	-1.11	.27	-.23	-.65	.18

^a^Levene’s test for homogeneity of variance was significant and therefore Satterthwaite approximation of *df* was used.* *p* < .05.

NB: Samples included non-binary/third gender participants (kidney transplant recipient group *n* = 2, comparison group *n* = 3) that could not be included in this analysis. Subscale key: UPE = *Unconditional Permission to Eat*; EPR = *Eating for Physical rather than emotional Reasons;* RHSC = *Reliance on Hunger and Satiety Cues*; BFCC = *Body-Food Choice Congruence.*

#### 4.2.4 Confirmatory factor analyses.

Eight confirmatory factor analyses were then carried out, two for each model outlined in Section 4.1.4 (i.e., with and without correlated errors). See Table 7 for fit indices and the S3 Tables for factor loadings for all identified models. As expected in a sample of this size, the chi-square tests for all models were highly significant in both the kidney transplant recipient and comparison groups.

**Table 7:** Model fit indices for confirmatory factor analyses.

	Kidney transplant recipients								Comparison group							
							RMSEA: 90% CIs								RMSEA: 90% CIs	
	χ2	*df*	CFI	TLI	SRMR	RMSEA	Lower	Upper	χ2	*df*	CFI	TLI	SRMR	RMSEA	Lower	Upper
Model 1	751.60	226	0.80	0.78	0.13	0.10	0.09	0.11	691.47	226	0.85	0.83	0.13	0.09	0.09	0.10
Model 1-CE	619.27	215	0.85	0.82	0.12	0.09	0.08	0.10	569.44	215	0.88	0.86	0.13	0.08	0.07	0.09
Model 2	708.10	224	0.82	0.79	0.12	0.09	0.09	0.10	658.55	224	0.86	0.84	0.11	0.09	0.08	0.10
Model 2-CE	577.73	213	0.86	0.84	0.11	0.08	0.08	0.09	534.25	213	0.90	0.88	0.11	0.08	0.07	0.09
Model 3	474.73	215	0.90	0.89	0.07	0.07	0.06	0.08	549.58	215	0.90	0.87	0.10	0.08	0.07	0.09
Model 3-CE	374.59	204	0.94	0.92	0.07	0.06	0.05	0.07	454.87	204	0.92	0.90	0.10	0.07	0.06	0.08
Model 4	86.31	41	0.97	0.96	0.05	0.07	0.05	0.09	89.22	41	0.97	0.96	0.05	0.07	0.05	0.09

Models ending -CE indicate those with correlated errors. CFI = Comparative Fit Index; TLI = Tucker-Lewis Index; SRMR = Standardized Root Mean square Residual; RMSEA = Root Mean Square Error of Approximation.

**Model 1 and 1-CE.** This model was a hierarchical model consisting of 23 items loading onto four latent variables (‘Unconditional permission to eat’, ‘Eating for physical rather than emotional reasons’, ‘Reliance on hunger and satiety cues’ and ‘Body-food choice congruence’) which then loaded onto a higher-order latent variable of total intuitive eating. Inspection of fit indices showed that both versions of this model were a poor fit to the data; whilst the correlated errors model had slightly better fit, it was still inadequate according to the cutoffs outlined in Section 4.1.4. Inspection of factor loadings revealed problems with the subscales loading onto the global intuitive eating factor; in the kidney transplant recipient group, the ‘Reliance on hunger and satiety cues’ and ‘Unconditional permission to eat’ subscales failed to load significantly onto the global intuitive eating factor and in the comparison group all subscales failed to load significantly onto the global factor. This was the case in both models (with and without correlated errors). There were also several items that failed to load significantly onto their respective factor; in particular, within the kidney transplant recipient group, IES-04 (“*I get mad at myself for eating something unhealthy”*) failed to load onto the ‘Unconditional permission to eat’ subscale in both models (i.e., with and without correlated errors) and IES-09 (“*I have forbidden foods that I don’t allow myself to eat”*) failed to load onto the ‘Unconditional permission to eat’ subscale for this group in the model without correlated errors. IES-11 (“*I find myself eating when I am stressed out, even when I’m not physically hungry”*) also failed to load significantly onto the ‘Eating for physical rather than emotional reasons’ subscale in the comparison group for the model without correlated errors.

**Model 2 and 2-CE.** This model consisted of 23 items loading onto four correlated latent variables (‘Unconditional permission to eat’, ‘Eating for physical rather than emotional reasons’, ‘Reliance on hunger and satiety cues’ and ‘Body-food choice congruence’). Inspection of fit indices showed that both versions of this model were a poor fit to the data; again, whilst the correlated errors model had slightly better fit, it was still inadequate according to the cutoffs outlined in Section 4.1.4. Inspection of factor loadings showed that, as with the first model, IES-04 and IES-09 failed to load significantly onto the ‘Unconditional permission to eat’ factor in the kidney transplant group for both models (i.e., with and without correlated errors).

**Model 3 and 3-CE.** This model consisted of 23 items loading onto six latent variables (‘Avoiding forbidden foods’, ‘Permission to eat’, ‘Avoiding emotional eating’, ‘Avoiding food-related coping strategies’, ‘Reliance on hunger and satiety cues’ and ‘Body-food choice congruence’). Inspection of fit indices showed that the version of this model without correlated errors was a poor fit to the data but that the correlated errors version showed acceptable fit. Inspection of factor loadings showed that, as with the first three models, IES-09 failed to load significantly onto its respective factor (‘Avoiding forbidden foods’) in the kidney transplant recipient group for both models.

**Model 4 and 4-CE.** This model consisted of 11 items loading onto three latent variables (‘Eating for physical rather than emotional reasons’, ’Reliance on hunger and satiety cues’ and ‘Body-food choice congruence’). Inspection of fit indices showed that the version of this model without correlated errors was an excellent fit to the data, whilst the version with correlated errors did not identify. Inspection of factor loadings showed that all items loaded significantly onto their respective factors.

### 4.3 Discussion

The aim of the survey workstream was to assess the factorial and construct validity, and the internal consistency, of the IES-2 in a sample of kidney transplant recipients and a comparison group of people without kidney disease. This was achieved through item analysis, validity testing and finally a series of confirmatory factor analyses.

With the exception of the ‘Unconditional permission to eat’ subscale, which is explored in more detail below, relatively few problems were identified with the IES-2 during item analysis. Construct validity (both convergent and discriminant) were also largely supported, as the IES-2 and its subscales were generally positively associated with body appreciation and negatively associated with anxiety for both the kidney transplant recipient and comparison groups, as hypothesised. Total IES-2 and subscale scores were also unrelated to social desirability, which supports discriminant construct validity, but as the internal consistency of the scale used to measure social desirability was poor for both the kidney transplant recipient and comparison groups, these findings should be interpreted cautiously. Known groups validity was largely unsupported; although mean IES-2 and subscale scores were generally higher for men than women, which would be expected, these differences were not found to be statistically significant other than for ‘Eating for physical rather than emotional reasons’ subscale scores in both the kidney transplant recipient and comparison groups. This may be due to the unequal representation of men and women in both groups; women formed 59.2% of the kidney transplant recipient sample and 88.4% of the comparison sample, which may have led to the test having insufficient power to detect statistically significant differences between genders. Internal consistency was good in both samples for total IES-2 scores and all subscales apart from ‘Unconditional permission to eat’.

#### 4.3.1 ‘Unconditional permission to eat’ subscale.

The findings of this workstream overwhelmingly suggest that there are problems with the ‘Unconditional permission to eat’ subscale of the IES-2 in its current form. Firstly, item analysis identified item discrimination problems in the form of low item-total correlations for items in this subscale in both the kidney transplant recipient and comparison groups. Item validity problems were also identified for all items in the ‘Unconditional permission to eat’ subscale for the kidney transplant recipient group, again in the form of consistently low correlations. Three items from the ‘Unconditional permission to eat’ subscale also had problems with response distribution, in that some adjacent scale points shared <20% of responses.

Validity tests again pointed to problems with the ‘Unconditional permission to eat’ subscale. Convergent construct validity was largely supported, in that total intuitive eating and the four subscales were predominantly associated with the hypothesised constructs of body appreciation and anxiety in both the kidney transplant recipient and comparison groups. However, ‘Unconditional permission to eat’ was not significantly associated with body appreciation for kidney transplant recipients and was not significantly associated with anxiety for the comparison group. Internal consistency for the ‘Unconditional permission to eat’ subscale was also poor for both the kidney transplant recipient and comparison groups.

#### 4.3.2 Factorial validity.

**Fig 1:**
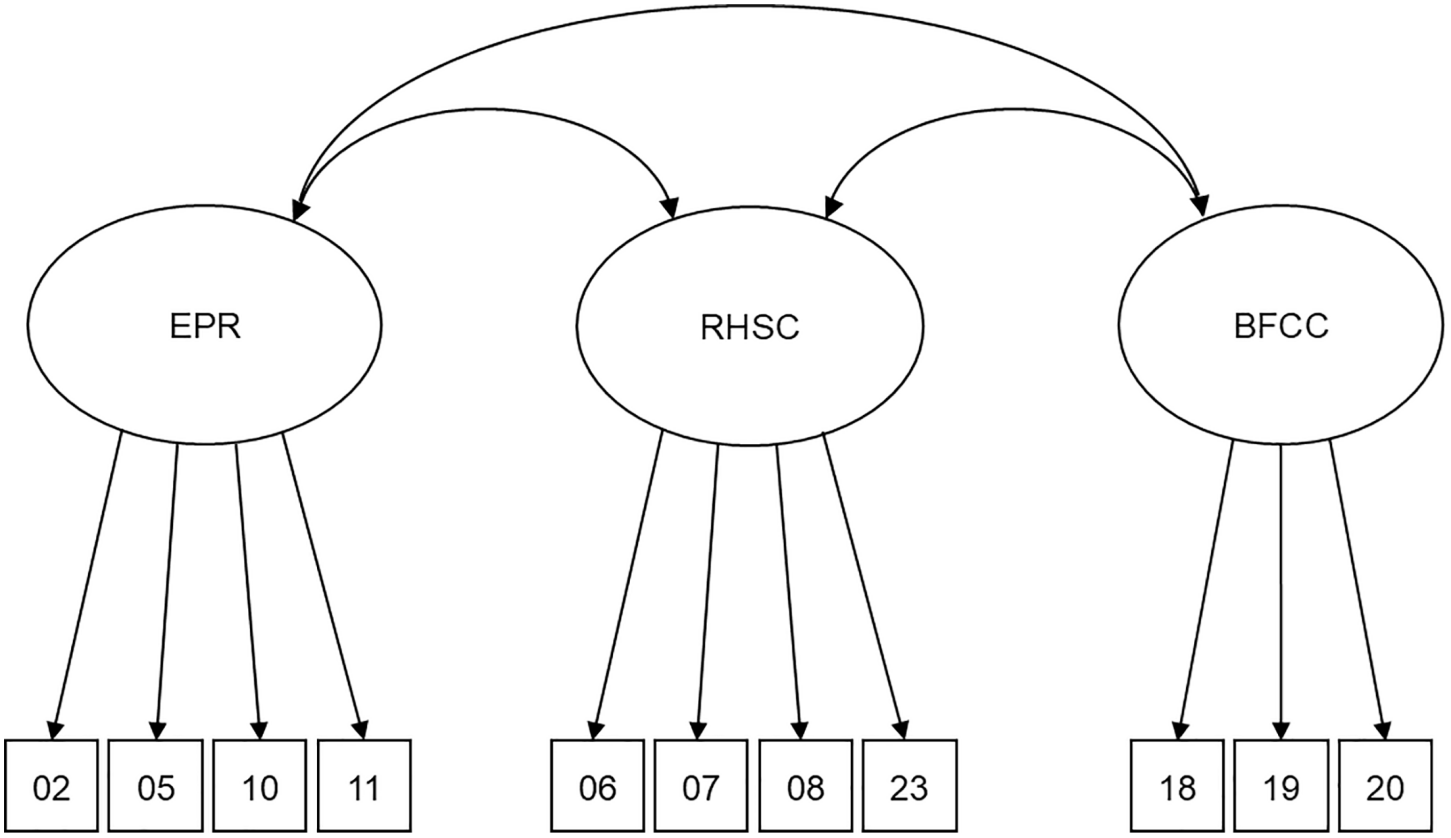
3-factor, 11-item model of the IES-2 (Saunders et al., 2018).

Confirmatory factor analyses found that the model presented in the original development and validation paper of the IES-2 [Model 1; [18] was a very poor fit to the data for both the kidney transplant recipient and comparison groups, as was Model 2 [56,57]. Model 3 [59], which used all of the original IES-2 items but arranged them into a six-factor model, showed acceptable fit to the data when the errors outlined in Section 4.1.4 were correlated to control for method effects. However, the model that showed the best fit to the data for both kidney transplant recipients and the comparison group was Model 4 [60], which only used 11 of the IES-2 items and arranged them into three correlated factors (see Fig 1). Crucially, this model does not include the ‘Unconditional permission to eat’ subscale which both workstreams in this study have identified as being so problematic, which may go some way in accounting for why this model fits the data so well in these samples. Inspection of factor loadings showed that two items from the ‘Unconditional permission to eat’ subscale – IES-04 (“*I get mad at myself for eating something unhealthy”*) and IES-09 (“*I have forbidden foods that I don’t allow myself to eat”*) – failed to load significantly on their respective factors in several of the models tested, highlighting again the potential problems with this factor and its items.

One reason why Saunders et al.’s [60] 11-item model may have fit the data better than the other models is because more complex models often fit the data worse due to more parameters meaning that there are more ways for the models to misfit. Shorter models naturally tend to fit better on standard fit indices because there are fewer estimated parameters and degrees of freedom are higher relative to chi-square. However, Saunders et al. [60] also demonstrated that their three-factor model was theoretically meaningful as well as statistically efficient, capturing clinically meaningful dimensions. There is also empirical support for the model from the present study and a more recent study in a Hispanic-majority sample [68]. The three factors preserved in this model cover the most critical intuitive eating processes; the subscale that was dropped (‘Unconditional permission to eat’) had weak psychometric performance in a number of samples, suggesting that its removal improves validity as well as statistical fit. Finally, a short, valid version of the scale is highly valuable in clinical settings, particularly where respondent burden must be minimised.

#### 4.3.3 Strengths and limitations.

A major methodological strength of this study is its concurrent triangulation mixed-methods design, in which qualitative and quantitative workstreams were conducted in parallel rather than sequentially. This approach enabled immediate cross-validation between participants’ lived experiences and the psychometric properties of the IES-2, providing a richer and more reliable evaluation than could have been achieved by either method alone. The parallel design also reduced the risk of bias introduced by sequencing, as neither workstream influenced the other during data collection. By integrating qualitative interpretation with quantitative analysis in real time, the study reflects the complex, dynamic nature of intuitive eating transitions among kidney transplant recipients. Moreover, this methodological strategy ensures that the refined IES-2 structure is not only statistically optimised but also grounded in authentic participant understanding, thereby enhancing both ecological and construct validity. Future validation research, particularly in clinical and transitional populations, may benefit from adopting similar concurrent triangulation approaches to optimise the balance between methodological rigour and contextual sensitivity.

Another main strength of this workstream is its consideration of several different factor structures of the IES-2 beyond the one presented by the original authors. Rather than simply testing the original model and assessing whether or not it fitted, this workstream considered alternative factor structures to explore whether these were a better fit to the data. As a result, this workstream has identified that the 11-item, three-factor model presented by Saunders et al. [60] shows superior fit to the data for both kidney transplant recipients and the comparison group in comparison to both the original model and the other models tested.

One potential limitation of the present workstream is the sociodemographic characteristics of the samples. Both the kidney transplant recipient and comparison groups were predominantly female and white, and the education level of both groups was also higher than the national average, as more than 70% of the kidney transplant recipient group and more than 86% of the comparison group had completed some form of Higher Education compared to 42% of working age people in the UK as of September 2017 [69]. This may be due to the use of social media to recruit to the study, as participants recruited in this way are more likely to be female and of a higher socioeconomic status [70]. This means that the findings of this workstream may not be generalisable to other samples with different sociodemographic characteristics, particularly given known cultural and socioeconomic variability in dietary habits and approaches to food [71–74]. There is likely to be variation in how people from different cultures and socioeconomic statuses respond to pre-transplant food restrictions and subsequent removal of restrictions post-transplant; therefore further research is needed to explore these findings in more depth and ascertain their applicability across other more ethnically and socioeconomically diverse transplant populations, including Asian or mixed-ethnicity samples. There were also some small differences between the sociodemographic characteristics of the kidney transplant recipient and comparison groups. However, addressing demographic differences in psychometric validation typically involves conducting measurement invariance testing, which was beyond the scope of the current study. The demographic differences do not invalidate the psychometric analyses presented but the authors acknowledge that the lack of invariance testing is a limitation of the present study. Future studies may benefit from examining measurement invariance across sociodemographic subgroups.

## 5 Overall conclusion

The aim of this study was to assess the extent to which the IES-2 is a valid and reliable measure of intuitive eating for kidney transplant recipients. Workstreams A and B were conducted concurrently to enable a concurrent triangulation mixed-methods approach, facilitating immediate cross-validation of qualitative and quantitative findings and enhancing the robustness of the validation process. The face validity of the IES-2 was assessed using thinkaloud interviews with kidney transplant recipients (Workstream A) and the factorial and construct validity were then assessed using a quantitative survey, along with the internal consistency reliability of the scale (Workstream B). This survey also enabled comparisons of reliability and validity to be drawn between kidney transplant recipients and a comparison group of people without kidney disease.

The findings of Workstream A and B were mixed using a weaving approach [26], which allowed for an overall impression of the validity of the IES-2 to be formed. For example, the quantitative analysis found that there were several psychometric problems with the ‘Unconditional permission to eat’ subscale, and possible reasons for this were illuminated by the qualitative findings, which showed that responses to the questions in this subscale were frequently affected by food restrictions related to anti-rejection medication or immunocompromisation and kidney-specific measures they needed to take to ensure the longevity of their transplant. With the exception of the ‘Unconditional permission to eat’ subscale (discussed below), the findings of this study suggest that the IES-2 can still be a useful tool for use with kidney transplant recipients despite there being some problems highlighted with the other subscales. Beyond the specific case of kidney transplant recipients, the findings also suggest broader implications for intuitive eating research. Other clinical populations, such as individuals living with diabetes, coeliac disease, or post-bariatric surgery, may similarly experience medically necessary dietary limitations that alter intuitive eating behaviours. Validation studies such as this one are therefore crucial in critically assessing the universality of intuitive eating constructs across diverse populations. Moreover, the integration of cognitive interviewing and confirmatory factor analysis in this study offers a methodological blueprint for future validation efforts in clinical contexts. By combining participant-led cognitive data with robust psychometric testing, researchers can more comprehensively evaluate scale suitability in populations with specific healthcare needs.

### 5.1 ‘Unconditional permission to eat’ subscale

Both of the workstreams highlighted significant issues with the ‘Unconditional permission to eat’ subscale of the IES-2, suggesting that this dimension of the scale may be less relevant for kidney transplant recipients or incompatible with their self-management. The thinkaloud interviews in Workstream A found that items in this subscale were often affected by the respondent being a kidney transplant recipient as their approach to ‘forbidden’ foods and eating plans is coloured by needing to consider avoiding certain foods due to immunocompromisation and medication restrictions. The survey in Workstream B also highlighted psychometric problems with this subscale. Interestingly, some of these were not confined to just the kidney transplant recipient group but extended to the comparison group too, suggesting that this subscale may not be psychometrically as sound overall.

### 5.3 The Intuitive Eating Scale-3

At time of writing, the Intuitive Eating Scale-3 (IES-3) has recently been published [24]. As outlined in Section 3.3, this version of the scale has sought to address some of the issues with the IES-2 that were identified by the participants in this study. For example, clearer instructions have been given to participants who need to avoid certain foods due to medical issues, allergies or religious observances, and the wording of the scale’s items are less repetitive and therefore less likely to cause confusion. As the IES-3’s items are novel, it will not be possible to draw conclusions about the performance of the new scale in samples of kidney transplant recipients based on the findings of this study as the items are likely to perform differently from a psychometric perspective. Crucially, however, the IES-3 has not been validated in any clinical samples to date and therefore the findings from this study remain highly relevant as the only known validation of a scale of intuitive eating for use with kidney transplant recipients.

### 5.4 Final recommendations

Given the problems identified with the ‘Unconditional permission to eat’ subscale, the IES-2 in its current form does not appear to be reliable or valid for use with kidney transplant recipients, and results should be interpreted judiciously if this dimension of the IES-2 is used. A preferable solution would be to use the alternative model of the IES-2 espoused by Saunders et al. [60] which presents a three-factor, 11-item model which does not incorporate the ‘Unconditional permission to eat’ subscale or the items it contains. Given the excellent level of fit to the data that this model provided in Workstream B, this model appears to be a good representation of intuitive eating within this sample and offers a way forward for the scale’s continued use.. This study’s identification of a valid and reliable measure of intuitive eating for kidney transplant recipients means that multidisciplinary transplant clinics could now integrate the assessment of intuitive eating into their treatment approaches, which could help them identify and address emotional eating patterns, medication-induced appetite changes and lifestyle transitions after transplantation in the patients that they are seeing. The scale could also be used in nutritional counselling – particularly as it has been suggested that nutritional counselling using intuitive eating as a concept may improve adherence to dietary recommendations for people with chronic kidney disease [23]. It may also be beneficial to integrate it into self-management programmes such as ‘My Kidneys and Me’ [75], which provide tailored information and support to people with chronic kidney disease.

Although, as outlined in Sections 3.3 and 5.3, there is now a newer version of the Intuitive Eating Scale [24] that may have addressed some of the problems identified in Workstream A, it is not possible to predict how valid and reliable this newer scale will be for kidney transplant recipients and therefore it is recommended that until a rigorous validation of the scale has been undertaken for this group, the IES-2 is used with the modifications outlined in this paper.

## Supporting information

S1 Table IES-2 problems identified during thinkaloud interviews. (DOCX)

S2 Table Item facility indices and item-total correlations for IES-2. (DOCX)

S3 Table CFA factor loadings. (DOCX)
