# Supplementary material for: A mixed-methods validation of the Intuitive Eating Scale-2 for use with kidney transplant recipients
Source: PLoS One. 2026 Jan 21;21(1):e0340998. doi: 10.1371/journal.pone.0340998 (PMC12822964; doi:10.1371/journal.pone.0340998)
Supplement: S1 Table — (DOCX) [file pone.0340998.s001.docx]

| **S1 Table** |  |  |  | |  | |  | |  | |  | |  | |  | |  | |
| --- | --- | --- | --- | --- | --- | --- | --- | --- | --- | --- | --- | --- | --- | --- | --- | --- | --- | --- |
| IES-2 problems identified during thinkaloud interviews | | | | | | | | | | | | | | | | | | |
|  | No problems  n (%) | Re-read / stumbled reading | Difficulty generating an answer | | Difficulty with response format | | Questioned content | | Confusion or mis-interpreted | | Not relevant to participant | | Answer affected by CKD | | Other | | Total problems  n (%)^a^ | |
| **Unconditional Permission to Eat** |  |  |  | |  | |  | |  | |  | | 1.5 | |  | |  | |
| I try to avoid certain foods high in fat, carbohydrates, or calories | 7 (77.8) | 1 | 1 | |  | | 2 | |  | |  | |  | |  | | 4 (5.6) | |
| If I am craving a certain food, I allow myself to have it | 9 (100.0) |  |  | |  | |  | |  | |  | |  | |  | | - | |
| I get mad at myself for eating something unhealthy | 7 (77.8) |  | 1 | |  | |  | |  | |  | | 1 | |  | | 2 (2.8) | |
| I have forbidden foods that I don’t allow myself to eat | 3 (33.3) |  | 1 | |  | | 1 | |  | |  | | 4 | |  | | 6 (8.3) | |
| I allow myself to eat what food I desire at the moment | 5 (55.6) |  |  |  | | 3 | |  | |  | | 1 | |  | | 4 (5.6) | |  |
| I do not follow eating rules or dieting plans that dictate what, when and/or how much to eat | 6 (66.7) |  | 1 |  | | 1 | |  | |  | | 3 | |  | | 5 (6.9) | |  |
|  |  |  |  |  | |  | |  | |  | |  | |  | |  | |  |
| **Eating for Physical rather than Emotional Reasons** |  |  |  |  | |  | |  | |  | | 0.63 | |  | |  | |  |
| I find myself eating when I’m feeling emotional (e.g. anxious, depressed, sad), even when I’m not physically hungry | 7 (77.8) |  |  | |  | |  | |  | | 1 | | 1 | |  | | 2 (2.8) | |
| I find myself eating when I am lonely, even when I’m not physically hungry | 3 (33.3) | 1 | 1 | |  | |  | |  | | 2 | | 2 | |  | | 6 (8.3) | |
| I use food to help me soothe my negative emotions | 7 (77.8) |  |  | |  | | 1 | | 1 | | 1 | |  | |  | | 3 (4.2) | |
| I find myself eating when I am stressed out, even when I’m not physically hungry | 7 (77.8) |  |  | |  | | 2 | |  | |  | |  | |  | | 2 (2.8) | |
|  | No problems  n (%) | Re-read / stumbled reading | Difficulty generating an answer | | Difficulty with response format | | Questioned content | | Confusion or mis-interpreted | | Not relevant to participant | | Answer affected by CKD | | Other | | Total problems  n (%)^a^ | |
| I am able to cope with my negative emotions (e.g. anxiety, sadness) without turning to food for comfort | 9 (100.0) |  |  | |  | |  | |  | |  | |  | |  | | - | |
| When I am bored, I do not eat just for something to do | 5 (55.6) | 2 | 1 | | 2 | | 1 | |  | |  | |  | |  | | 6 (8.3) | |
| When I am lonely, I do not turn to food for comfort | 3 (33.3) |  | 1 | |  | | 2 | |  | | 2 | | 2 | |  | | 7 (9.7) | |
| I find other ways to cope with stress and anxiety than by eating | 7 (77.8) |  |  | | 1 | | 1 | |  | |  | |  | |  | | 2 (2.8) | |
| **Reliance on Hunger and Satiety Cues** |  |  |  | |  | |  | |  | |  | | 1.33 | |  | |  | |
| I trust my body to tell me when to eat | 8 (88.9) |  |  | |  | |  | |  | |  | | 1 | |  | | 1 (1.4) | |
| I trust my body to tell me what to eat | 5 (55.6) |  |  | |  | | 2 | |  | |  | | 2 | |  | | 4 (5.6) | |
| I trust my body to tell me how much to eat | 7 (77.8) |  |  | |  | | 1 | |  | |  | | 2 | |  | | 3 (4.2) | |
| I rely on my hunger signals to tell me when to eat | 8 (88.9) |  |  | |  | |  | |  | |  | | 1 | |  | | 1 (1.4) | |
| I rely on my fullness (satiety) signals to tell me when to stop eating | 6 (66.7) | 1 |  | |  | | 1 | |  | |  | | 2 | |  | | 4 (5.6) | |
| I trust my body to tell me when to stop eating | 7 (77.8) |  |  | |  | | 2 | |  | |  | |  | |  | | 2 (2.8) | |
|  |  |  |  | |  | |  | |  | |  | |  | |  | |  | |
| **Body-Food Choice Congruence** |  |  |  | |  | |  | |  | |  | | 1 | |  | |  | |
| Most of the time, I desire to eat nutritious foods | 9 (100.0) |  |  | |  | |  | |  | |  | |  | |  | | - | |
| I mostly eat foods that make my body perform efficiently (well) | 6 (66.7) |  |  | |  | | 1 | |  | |  | | 2 | |  | | 3 (4.2) | |
| I mostly eat foods that give my body energy and stamina | 5 (55.6) |  |  | | 1 | | 2 | |  | |  | | 1 | | 1 | | 5 (6.9) | |
| ^a^ Percentage of total problems identified (n = 72). CKD = Chronic kidney disease. | | | | | | | | | | | | | | | | | | |
