# Supplementary material for: A mixed-methods validation of the Intuitive Eating Scale-2 for use with kidney transplant recipients
Source: PLoS One. 2026 Jan 21;21(1):e0340998. doi: 10.1371/journal.pone.0340998 (PMC12822964; doi:10.1371/journal.pone.0340998)
Supplement: S2 Table — (DOCX) [file pone.0340998.s002.docx]

| **S2 Table** |  | |  | |
| --- | --- | --- | --- | --- |
| Item facility indices and item-total correlations for IES-2 | | | | |
|  | Item facility index | | Item-total *r* | |
|  | KTRs | Controls | KTRs | Controls |
| UPE |  |  |  |  |
| IES01 | 2.71 | 3.00 | -0.117^†^ | 0.048^†^ |
| IES03 | 3.58 | 3.83 | -0.046^†^ | 0.061^†^ |
| IES04 | 3.20 | 3.09 | 0.520 | 0.585 |
| IES09 | 3.35 | 3.89 | 0.034^†^ | 0.168^†^ |
| IES16 | 3.36 | 3.50 | 0.216^†^ | 0.222^†^ |
| IES17 | 3.49 | 3.49 | 0.226 | 0.356 |
| EPR |  |  |  |  |
| IES02 | 3.08 | 2.47 | 0.560 | 0.550 |
| IES05 | 3.46 | 3.10 | 0.606 | 0.547 |
| IES10 | 3.56 | 2.90 | 0.625 | 0.578 |
| IES11 | 3.38 | 2.83 | 0.629 | 0.601 |
| IES12 | 3.50 | 3.20 | 0.549 | 0.557 |
| IES13 | 3.31 | 2.87 | 0.511 | 0.491 |
| IES14 | 3.36 | 3.02 | 0.583 | 0.543 |
| IES15 | 3.60 | 3.60 | 0.606 | 0.615 |
| RHSC |  |  |  |  |
| IES06 | 3.18 | 3.12 | 0.443 | 0.645 |
| IES07 | 2.67 | 2.77 | 0.463 | 0.459 |
| IES08 | 2.96 | 2.96 | 0.576 | 0.657 |
| IES21 | 3.03 | 3.08 | 0.486 | 0.577 |
| IES22 | 3.21 | 3.22 | 0.535 | 0.553 |
| IES23 | 3.13 | 3.13 | 0.619 | 0.643 |
| BFCC |  |  |  |  |
| IES18 | 3.53 | 3.45 | 0.408 | 0.361 |
| IES19 | 3.30 | 3.16 | 0.483 | 0.419 |
| IES20 | 3.28 | 3.23 | 0.480 | 0.428 |
| ^†^ item-total correlations < 0.3. KTRs = Kidney transplant recipients.  UPE = *Unconditional permission to eat*  EPR = *Eating for physical rather than emotional reasons* RHSC = *Reliance on hunger and satiety cues* BFCC = *Body-food choice congruence* | | | | |
