# Supplementary material for: A mixed-methods validation of the Intuitive Eating Scale-2 for use with kidney transplant recipients
Source: PLoS One. 2026 Jan 21;21(1):e0340998. doi: 10.1371/journal.pone.0340998 (PMC12822964; doi:10.1371/journal.pone.0340998)
Supplement: S3 Table — (DOCX) [file pone.0340998.s003.docx]

| **S3 Table A** | | | | | |
| --- | --- | --- | --- | --- | --- |
| CFA factor loadings for Model 1: Kidney transplant recipient sample | | | | | |
| Factor | Indicator | Item | *B* | *β* | *R^2^* |
| Global | RHSC |  | 1.00 | 0.56 | 0.32 |
|  | UPE |  | -0.05 | -0.05 | 0.00 |
|  | EPR |  | 2.17*** | 0.81 | 0.65 |
|  | BFCC |  | 1.11*** | 0.56 | 0.31 |
| UPE | IES_01 | I try to avoid certain foods high in fat, carbohydrates, or calories | 1.00 | 0.37 | 0.13 |
|  | IES_04 | I get mad at myself for eating something unhealthy | 0.09 | 0.02 | 0.00 |
|  | IES_09 | I have forbidden foods that I don’t allow myself to eat | 0.89 | 0.22 | 0.05 |
|  | IES_03 | If I am craving a certain food, I allow myself to have it | 1.73*** | 0.58 | 0.34 |
|  | IES_16 | I allow myself to eat what food I desire at the moment | 2.27*** | 0.80 | 0.63 |
|  | IES_17 | I do not follow eating rules or dieting plans that dictate what, when and/or how much to eat | 1.42*** | 0.44 | 0.19 |
| EPR | IES_02 | I find myself eating when I’m feeling emotional, even when I’m not physically hungry | 1.00 | 0.81 | 0.65 |
|  | IES_11 | I find myself eating when I am stressed out, even when I’m not physically hungry | 1.10*** | 0.89 | 0.78 |
|  | IES_12 | I am able to cope with my negative emotions (e.g. anxiety, sadness) without turning to food for comfort | 0.65*** | 0.60 | 0.36 |
|  | IES_13 | When I am bored, I do not eat just for something to do | 0.58*** | 0.53 | 0.28 |
|  | IES_14 | When I am lonely, I do not turn to food for comfort | 0.72*** | 0.62 | 0.39 |
|  | IES_15 | I find other ways to cope with stress and anxiety than by eating | 0.64*** | 0.61 | 0.38 |
|  | IES_10 | I use food to help me soothe my negative emotions | 0.99*** | 0.87 | 0.75 |
|  | IES_05 | I find myself eating when I am lonely, even when I’m not physically hungry | 0.94*** | 0.80 | 0.65 |
| RHSC | IES_06 | I trust my body to tell me when to eat | 1.00 | 0.63 | 0.39 |
|  | IES_08 | I trust my body to tell me how much to eat | 1.23*** | 0.77 | 0.59 |
|  | IES_21 | I rely on my hunger signals to tell me when to eat | 1.01*** | 0.64 | 0.41 |
|  | IES_07 | I trust my body to tell me what to eat | 0.92*** | 0.60 | 0.36 |
|  | IES_22 | I rely on my fullness (satiety) signals to tell me when to stop eating | 1.17*** | 0.72 | 0.52 |
|  | IES_23 | I trust my body to tell me when to stop eating | 1.33*** | 0.79 | 0.62 |
| BFCC | IES_18 | Most of the time, I desire to eat nutritious foods | 1.00 | 0.76 | 0.58 |
|  | IES_19 | I mostly eat foods that make my body perform efficiently (well) | 1.23*** | 0.97 | 0.94 |
|  | IES_20 | I mostly eat foods that give my body energy and stamina | 0.93*** | 0.77 | 0.59 |
| *** Significant at p <.001. *B* = Unstandardised factor loadings, *β =* Standardised factor loadings.  UPE = Unconditional permission to eat; EPR: Eating for physical rather than emotional reasons; RHSC = Reliance on hunger & satiety cues; BFCC = Body-food choice congruence | | | | | |

| **S3 Table B** | | | | | |
| --- | --- | --- | --- | --- | --- |
| CFA factor loadings for Model 1: Comparison group | | | | | |
| Factor | Indicator | Item | *B* | *β* | *R^2^* |
| Global | RHSC |  | 1.00 | 2.28 | 5.18 |
|  | UPE |  | 0.06 | 0.22 | 0.05 |
|  | EPR |  | 0.10 | 0.18 | 0.03 |
|  | BFCC |  | 0.06 | 0.14 | 0.02 |
| UPE | IES_01 | I try to avoid certain foods high in fat, carbohydrates, or calories | 1.00 | 0.51 | 0.26 |
|  | IES_04 | I get mad at myself for eating something unhealthy | 0.75*** | 0.36 | 0.13 |
|  | IES_09 | I have forbidden foods that I don’t allow myself to eat | 1.27*** | 0.58 | 0.34 |
|  | IES_03 | If I am craving a certain food, I allow myself to have it | 1.09*** | 0.68 | 0.46 |
|  | IES_16 | I allow myself to eat what food I desire at the moment | 1.16*** | 0.71 | 0.50 |
|  | IES_17 | I do not follow eating rules or dieting plans that dictate what, when and/or how much to eat | 1.35*** | 0.59 | 0.35 |
| EPR | IES_02 | I find myself eating when I’m feeling emotional, even when I’m not physically hungry | 1.00 | 0.83 | 0.69 |
|  | IES_11 | I find myself eating when I am stressed out, even when I’m not physically hungry | 1.11 | 0.89 | 0.79 |
|  | IES_12 | I am able to cope with my negative emotions (e.g. anxiety, sadness) without turning to food for comfort | 0.68*** | 0.67 | 0.45 |
|  | IES_13 | When I am bored, I do not eat just for something to do | 0.68*** | 0.56 | 0.32 |
|  | IES_14 | When I am lonely, I do not turn to food for comfort | 0.80*** | 0.68 | 0.46 |
|  | IES_15 | I find other ways to cope with stress and anxiety than by eating | 0.63*** | 0.67 | 0.44 |
|  | IES_10 | I use food to help me soothe my negative emotions | 0.98*** | 0.85 | 0.73 |
|  | IES_05 | I find myself eating when I am lonely, even when I’m not physically hungry | 0.90*** | 0.76 | 0.58 |
| RHSC | IES_06 | I trust my body to tell me when to eat | 1.00 | 0.74 | 0.55 |
|  | IES_08 | I trust my body to tell me how much to eat | 1.13*** | 0.82 | 0.67 |
|  | IES_21 | I rely on my hunger signals to tell me when to eat | 0.85*** | 0.70 | 0.48 |
|  | IES_07 | I trust my body to tell me what to eat | 0.76*** | 0.58 | 0.34 |
|  | IES_22 | I rely on my fullness (satiety) signals to tell me when to stop eating | 0.96*** | 0.75 | 0.56 |
|  | IES_23 | I trust my body to tell me when to stop eating | 1.11*** | 0.82 | 0.67 |
| BFCC | IES_18 | Most of the time, I desire to eat nutritious foods | 1.00 | 0.76 | 0.58 |
|  | IES_19 | I mostly eat foods that make my body perform efficiently (well) | 1.25*** | 0.97 | 0.94 |
|  | IES_20 | I mostly eat foods that give my body energy and stamina | 1.06*** | 0.88 | 0.77 |
| *** Significant at p <.001. *B* = Unstandardised factor loadings, *β =* Standardised factor loadings.  UPE = Unconditional permission to eat; EPR: Eating for physical rather than emotional reasons; RHSC = Reliance on hunger & satiety cues; BFCC = Body-food choice congruence | | | | | |

| **S3 Table C** | | | | | |
| --- | --- | --- | --- | --- | --- |
| CFA factor loadings for Model 1-CE: Kidney transplant recipient sample | | | | | |
| Factor | Indicator | Item | *B* | *β* | *R^2^* |
| Global | RHSC |  | 1.00 | 0.48 | 0.23 |
|  | UPE |  | -0.17 | -0.13 | 0.02 |
|  | EPR |  | 3.32*** | 1.05 | 1.09 |
|  | BFCC |  | 1.55*** | 0.52 | 0.27 |
| UPE | IES_01 | I try to avoid certain foods high in fat, carbohydrates, or calories | 1.00 | 0.39 | 0.15 |
|  | IES_04 | I get mad at myself for eating something unhealthy | -0.01 | 0.00 | 0.00 |
|  | IES_09 | I have forbidden foods that I don’t allow myself to eat | 0.85*** | 0.23 | 0.05 |
|  | IES_03 | If I am craving a certain food, I allow myself to have it | 1.69*** | 0.61 | 0.37 |
|  | IES_16 | I allow myself to eat what food I desire at the moment | 2.05*** | 0.76 | 0.58 |
|  | IES_17 | I do not follow eating rules or dieting plans that dictate what, when and/or how much to eat | 1.31*** | 0.43 | 0.18 |
| EPR | IES_02 | I find myself eating when I’m feeling emotional, even when I’m not physically hungry | 1.00 | 0.69 | 0.47 |
|  | IES_11 | I find myself eating when I am stressed out, even when I’m not physically hungry | 1.15*** | 0.78 | 0.61 |
|  | IES_12 | I am able to cope with my negative emotions (e.g. anxiety, sadness) without turning to food for comfort | 0.82*** | 0.64 | 0.41 |
|  | IES_13 | When I am bored, I do not eat just for something to do | 0.80*** | 0.62 | 0.39 |
|  | IES_14 | When I am lonely, I do not turn to food for comfort | 0.97*** | 0.72 | 0.51 |
|  | IES_15 | I find other ways to cope with stress and anxiety than by eating | 0.86*** | 0.71 | 0.50 |
|  | IES_10 | I use food to help me soothe my negative emotions | 1.10*** | 0.82 | 0.67 |
|  | IES_05 | I find myself eating when I am lonely, even when I’m not physically hungry | 1.01*** | 0.74 | 0.54 |
| RHSC | IES_06 | I trust my body to tell me when to eat | 1.00 | 0.53 | 0.28 |
|  | IES_08 | I trust my body to tell me how much to eat | 1.28*** | 0.67 | 0.45 |
|  | IES_21 | I rely on my hunger signals to tell me when to eat | 1.18*** | 0.63 | 0.40 |
|  | IES_07 | I trust my body to tell me what to eat | 0.86*** | 0.47 | 0.22 |
|  | IES_22 | I rely on my fullness (satiety) signals to tell me when to stop eating | 1.48*** | 0.77 | 0.59 |
|  | IES_23 | I trust my body to tell me when to stop eating | 1.76*** | 0.88 | 0.78 |
| BFCC | IES_18 | Most of the time, I desire to eat nutritious foods | 1.00 | 0.82 | 0.67 |
|  | IES_19 | I mostly eat foods that make my body perform efficiently (well) | 1.08*** | 0.91 | 0.83 |
|  | IES_20 | I mostly eat foods that give my body energy and stamina | 0.80*** | 0.70 | 0.49 |
| *** Significant at p <.001. *B* = Unstandardised factor loadings, *β =* Standardised factor loadings.  UPE = Unconditional permission to eat; EPR: Eating for physical rather than emotional reasons; RHSC = Reliance on hunger & satiety cues; BFCC = Body-food choice congruence | | | | | |

| **S3 Table D** | | | | | |
| --- | --- | --- | --- | --- | --- |
| CFA factor loadings for Model 1-CE: Comparison group | | | | | |
| Factor | Indicator | Item | *B* | *β* | *R^2^* |
| Global | RHSC |  | 1.00 | 2.16 | 4.66 |
|  | UPE |  | 0.08 | 0.22 | 0.05 |
|  | EPR |  | 0.12 | 0.20 | 0.04 |
|  | BFCC |  | 0.06 | 0.11 | 0.01 |
| UPE | IES_01 | I try to avoid certain foods high in fat, carbohydrates, or calories | 1.00 | 0.51 | 0.26 |
|  | IES_04 | I get mad at myself for eating something unhealthy | 0.73*** | 0.36 | 0.13 |
|  | IES_09 | I have forbidden foods that I don’t allow myself to eat | 1.29*** | 0.59 | 0.35 |
|  | IES_03 | If I am craving a certain food, I allow myself to have it | 1.09*** | 0.68 | 0.46 |
|  | IES_16 | I allow myself to eat what food I desire at the moment | 1.16*** | 0.71 | 0.50 |
|  | IES_17 | I do not follow eating rules or dieting plans that dictate what, when and/or how much to eat | 1.34*** | 0.59 | 0.34 |
| EPR | IES_02 | I find myself eating when I’m feeling emotional, even when I’m not physically hungry | 1.00 | 0.77 | 0.59 |
|  | IES_11 | I find myself eating when I am stressed out, even when I’m not physically hungry | 1.13*** | 0.84 | 0.70 |
|  | IES_12 | I am able to cope with my negative emotions (e.g. anxiety, sadness) without turning to food for comfort | 0.77*** | 0.70 | 0.49 |
|  | IES_13 | When I am bored, I do not eat just for something to do | 0.78*** | 0.60 | 0.36 |
|  | IES_14 | When I am lonely, I do not turn to food for comfort | 0.91*** | 0.71 | 0.50 |
|  | IES_15 | I find other ways to cope with stress and anxiety than by eating | 0.71*** | 0.70 | 0.49 |
|  | IES_10 | I use food to help me soothe my negative emotions | 1.06*** | 0.85 | 0.72 |
|  | IES_05 | I find myself eating when I am lonely, even when I’m not physically hungry | 0.93*** | 0.73 | 0.53 |
| RHSC | IES_06 | I trust my body to tell me when to eat | 1.00 | 0.67 | 0.45 |
|  | IES_08 | I trust my body to tell me how much to eat | 1.17*** | 0.76 | 0.57 |
|  | IES_21 | I rely on my hunger signals to tell me when to eat | 0.88*** | 0.65 | 0.43 |
|  | IES_07 | I trust my body to tell me what to eat | 0.68*** | 0.47 | 0.22 |
|  | IES_22 | I rely on my fullness (satiety) signals to tell me when to stop eating | 1.16*** | 0.81 | 0.66 |
|  | IES_23 | I trust my body to tell me when to stop eating | 1.40*** | 0.93 | 0.86 |
| BFCC | IES_18 | Most of the time, I desire to eat nutritious foods | 1.00 | 0.77 | 0.60 |
|  | IES_19 | I mostly eat foods that make my body perform efficiently (well) | 1.21*** | 0.95 | 0.91 |
|  | IES_20 | I mostly eat foods that give my body energy and stamina | 1.02*** | 0.85 | 0.73 |
| *** Significant at p <.001. *B* = Unstandardised factor loadings, *β =* Standardised factor loadings.  UPE = Unconditional permission to eat; EPR: Eating for physical rather than emotional reasons; RHSC = Reliance on hunger & satiety cues; BFCC = Body-food choice congruence | | | | | |

| **S3 Table E** | | | | | |
| --- | --- | --- | --- | --- | --- |
| CFA factor loadings for Model 2: Kidney transplant recipient sample | | | | | |
| Factor | Indicator | Item | *B* | *β* | *R^2^* |
| UPE | IES_01 | I try to avoid certain foods high in fat, carbohydrates, or calories | 1.00 | 0.40 | 0.16 |
|  | IES_04 | I get mad at myself for eating something unhealthy | -0.05 | -0.02 | 0.00 |
|  | IES_09 | I have forbidden foods that I don’t allow myself to eat | 0.78 | 0.22 | 0.05 |
|  | IES_03 | If I am craving a certain food, I allow myself to have it | 1.69*** | 0.63 | 0.39 |
|  | IES_16 | I allow myself to eat what food I desire at the moment | 1.92*** | 0.74 | 0.55 |
|  | IES_17 | I do not follow eating rules or dieting plans that dictate what, when and/or how much to eat | 1.22*** | 0.41 | 0.17 |
| EPR | IES_02 | I find myself eating when I’m feeling emotional, even when I’m not physically hungry | 1.00 | 0.81 | 0.65 |
|  | IES_11 | I find myself eating when I am stressed out, even when I’m not physically hungry | 1.10*** | 0.89 | 0.79 |
|  | IES_12 | I am able to cope with my negative emotions (e.g. anxiety, sadness) without turning to food for comfort | 0.65*** | 0.60 | 0.36 |
|  | IES_13 | When I am bored, I do not eat just for something to do | 0.57*** | 0.53 | 0.28 |
|  | IES_14 | When I am lonely, I do not turn to food for comfort | 0.71*** | 0.62 | 0.39 |
|  | IES_15 | I find other ways to cope with stress and anxiety than by eating | 0.63*** | 0.61 | 0.38 |
|  | IES_10 | I use food to help me soothe my negative emotions | 0.99*** | 0.87 | 0.75 |
|  | IES_05 | I find myself eating when I am lonely, even when I’m not physically hungry | 0.94*** | 0.80 | 0.65 |
| RHSC | IES_06 | I trust my body to tell me when to eat | 1.00 | 0.61 | 0.38 |
|  | IES_08 | I trust my body to tell me how much to eat | 1.25*** | 0.76 | 0.58 |
|  | IES_21 | I rely on my hunger signals to tell me when to eat | 1.03*** | 0.64 | 0.41 |
|  | IES_07 | I trust my body to tell me what to eat | 0.94*** | 0.60 | 0.36 |
|  | IES_22 | I rely on my fullness (satiety) signals to tell me when to stop eating | 1.21*** | 0.73 | 0.53 |
|  | IES_23 | I trust my body to tell me when to stop eating | 1.38*** | 0.80 | 0.64 |
| BFCC | IES_18 | Most of the time, I desire to eat nutritious foods | 1.00 | 0.76 | 0.58 |
|  | IES_19 | I mostly eat foods that make my body perform efficiently (well) | 1.23*** | 0.97 | 0.94 |
|  | IES_20 | I mostly eat foods that give my body energy and stamina | 0.93*** | 0.77 | 0.59 |
| *** Significant at p <.001. *B* = Unstandardised factor loadings, *β =* Standardised factor loadings.  UPE = Unconditional permission to eat; EPR: Eating for physical rather than emotional reasons; RHSC = Reliance on hunger & satiety cues; BFCC = Body-food choice congruence | | | | | |

| **S3 Table F** | | | | | |
| --- | --- | --- | --- | --- | --- |
| CFA factor loadings for Model 2: Comparison group | | | | | |
| Factor | Indicator | Item | *B* | *β* | *R^2^* |
| UPE | IES_01 | I try to avoid certain foods high in fat, carbohydrates, or calories | 1.00 | 0.52 | 0.27 |
|  | IES_04 | I get mad at myself for eating something unhealthy | 0.67*** | 0.34 | 0.11 |
|  | IES_09 | I have forbidden foods that I don’t allow myself to eat | 1.25*** | 0.58 | 0.34 |
|  | IES_03 | If I am craving a certain food, I allow myself to have it | 1.09*** | 0.69 | 0.48 |
|  | IES_16 | I allow myself to eat what food I desire at the moment | 1.13*** | 0.70 | 0.50 |
|  | IES_17 | I do not follow eating rules or dieting plans that dictate what, when and/or how much to eat | 1.28*** | 0.57 | 0.33 |
| EPR | IES_02 | I find myself eating when I’m feeling emotional, even when I’m not physically hungry | 1.00 | 0.83 | 0.68 |
|  | IES_11 | I find myself eating when I am stressed out, even when I’m not physically hungry | 1.11*** | 0.34 | 0.79 |
|  | IES_12 | I am able to cope with my negative emotions (e.g. anxiety, sadness) without turning to food for comfort | 0.69*** | 0.58 | 0.46 |
|  | IES_13 | When I am bored, I do not eat just for something to do | 0.68*** | 0.69 | 0.32 |
|  | IES_14 | When I am lonely, I do not turn to food for comfort | 0.81*** | 0.70 | 0.47 |
|  | IES_15 | I find other ways to cope with stress and anxiety than by eating | 0.64*** | 0.57 | 0.45 |
|  | IES_10 | I use food to help me soothe my negative emotions | 0.98*** | 0.34 | 0.72 |
|  | IES_05 | I find myself eating when I am lonely, even when I’m not physically hungry | 0.90*** | 0.58 | 0.58 |
| RHSC | IES_06 | I trust my body to tell me when to eat | 1.00 | 0.74 | 0.55 |
|  | IES_08 | I trust my body to tell me how much to eat | 1.14*** | 0.82 | 0.67 |
|  | IES_21 | I rely on my hunger signals to tell me when to eat | 0.85*** | 0.69 | 0.48 |
|  | IES_07 | I trust my body to tell me what to eat | 0.76*** | 0.58 | 0.34 |
|  | IES_22 | I rely on my fullness (satiety) signals to tell me when to stop eating | 0.96*** | 0.75 | 0.56 |
|  | IES_23 | I trust my body to tell me when to stop eating | 1.11*** | 0.82 | 0.67 |
| BFCC | IES_18 | Most of the time, I desire to eat nutritious foods | 1.00 | 0.75 | 0.57 |
|  | IES_19 | I mostly eat foods that make my body perform efficiently (well) | 1.28*** | 0.98 | 0.96 |
|  | IES_20 | I mostly eat foods that give my body energy and stamina | 1.06*** | 0.86 | 0.75 |
| *** Significant at p <.001. *B* = Unstandardised factor loadings, *β =* Standardised factor loadings.  UPE = Unconditional permission to eat; EPR: Eating for physical rather than emotional reasons; RHSC = Reliance on hunger & satiety cues; BFCC = Body-food choice congruence | | | | | |

| **S3 Table G** | | | | | |
| --- | --- | --- | --- | --- | --- |
| CFA factor loadings for Model 2-CE: Kidney transplant recipient sample | | | | | |
| Factor | Indicator | Item | *B* | *β* | *R^2^* |
| UPE | IES_01 | I try to avoid certain foods high in fat, carbohydrates, or calories | 1.00 | 0.41 | 0.17 |
|  | IES_04 | I get mad at myself for eating something unhealthy | 0.00 | 0.00 | 0.00 |
|  | IES_09 | I have forbidden foods that I don’t allow myself to eat | 0.78 | 0.22 | 0.05 |
|  | IES_03 | If I am craving a certain food, I allow myself to have it | 1.62*** | 0.61 | 0.37 |
|  | IES_16 | I allow myself to eat what food I desire at the moment | 1.89*** | 0.75 | 0.56 |
|  | IES_17 | I do not follow eating rules or dieting plans that dictate what, when and/or how much to eat | 1.23*** | 0.42 | 0.18 |
| EPR | IES_02 | I find myself eating when I’m feeling emotional, even when I’m not physically hungry | 1.00 | 0.70 | 0.49 |
|  | IES_11 | I find myself eating when I am stressed out, even when I’m not physically hungry | 1.14*** | 0.80 | 0.64 |
|  | IES_12 | I am able to cope with my negative emotions (e.g. anxiety, sadness) without turning to food for comfort | 0.79*** | 0.63 | 0.40 |
|  | IES_13 | When I am bored, I do not eat just for something to do | 0.77*** | 0.61 | 0.37 |
|  | IES_14 | When I am lonely, I do not turn to food for comfort | 0.93*** | 0.70 | 0.50 |
|  | IES_15 | I find other ways to cope with stress and anxiety than by eating | 0.83*** | 0.69 | 0.48 |
|  | IES_10 | I use food to help me soothe my negative emotions | 1.09*** | 0.83 | 0.69 |
|  | IES_05 | I find myself eating when I am lonely, even when I’m not physically hungry | 1.01*** | 0.75 | 0.56 |
| RHSC | IES_06 | I trust my body to tell me when to eat | 1.00 | 0.52 | 0.27 |
|  | IES_08 | I trust my body to tell me how much to eat | 1.31*** | 0.68 | 0.46 |
|  | IES_21 | I rely on my hunger signals to tell me when to eat | 1.19*** | 0.63 | 0.40 |
|  | IES_07 | I trust my body to tell me what to eat | 0.92*** | 0.50 | 0.25 |
|  | IES_22 | I rely on my fullness (satiety) signals to tell me when to stop eating | 1.49*** | 0.76 | 0.58 |
|  | IES_23 | I trust my body to tell me when to stop eating | 1.79*** | 0.89 | 0.79 |
| BFCC | IES_18 | Most of the time, I desire to eat nutritious foods | 1.00 | 0.81 | 0.65 |
|  | IES_19 | I mostly eat foods that make my body perform efficiently (well) | 1.10*** | 0.92 | 0.84 |
|  | IES_20 | I mostly eat foods that give my body energy and stamina | 0.81*** | 0.71 | 0.51 |
| *** Significant at p <.001. *B* = Unstandardised factor loadings, *β =* Standardised factor loadings.  UPE = Unconditional permission to eat; EPR: Eating for physical rather than emotional reasons; RHSC = Reliance on hunger & satiety cues; BFCC = Body-food choice congruence | | | | | |

| **S3 Table H** | | | | | |
| --- | --- | --- | --- | --- | --- |
| CFA factor loadings for Model 2-CE: Comparison group | | | | | |
| Factor | Indicator | Item | *B* | *β* | *R^2^* |
| UPE | IES_01 | I try to avoid certain foods high in fat, carbohydrates, or calories | 1.00 | 0.52 | 0.27 |
|  | IES_04 | I get mad at myself for eating something unhealthy | 0.67*** | 0.33 | 0.11 |
|  | IES_09 | I have forbidden foods that I don’t allow myself to eat | 1.26*** | 0.59 | 0.35 |
|  | IES_03 | If I am craving a certain food, I allow myself to have it | 1.08*** | 0.69 | 0.48 |
|  | IES_16 | I allow myself to eat what food I desire at the moment | 1.13*** | 0.70 | 0.49 |
|  | IES_17 | I do not follow eating rules or dieting plans that dictate what, when and/or how much to eat | 1.28*** | 0.57 | 0.33 |
| EPR | IES_02 | I find myself eating when I’m feeling emotional, even when I’m not physically hungry | 1.00 | 0.76 | 0.58 |
|  | IES_11 | I find myself eating when I am stressed out, even when I’m not physically hungry | 1.13*** | 0.83 | 0.69 |
|  | IES_12 | I am able to cope with my negative emotions (e.g. anxiety, sadness) without turning to food for comfort | 0.78*** | 0.71 | 0.50 |
|  | IES_13 | When I am bored, I do not eat just for something to do | 0.79*** | 0.61 | 0.37 |
|  | IES_14 | When I am lonely, I do not turn to food for comfort | 0.92*** | 0.72 | 0.51 |
|  | IES_15 | I find other ways to cope with stress and anxiety than by eating | 0.73*** | 0.71 | 0.50 |
|  | IES_10 | I use food to help me soothe my negative emotions | 1.05*** | 0.84 | 0.70 |
|  | IES_05 | I find myself eating when I am lonely, even when I’m not physically hungry | 0.93*** | 0.73 | 0.53 |
| RHSC | IES_06 | I trust my body to tell me when to eat | 1.00 | 0.66 | 0.44 |
|  | IES_08 | I trust my body to tell me how much to eat | 1.17*** | 0.75 | 0.57 |
|  | IES_21 | I rely on my hunger signals to tell me when to eat | 0.89*** | 0.65 | 0.43 |
|  | IES_07 | I trust my body to tell me what to eat | 0.68*** | 0.46 | 0.21 |
|  | IES_22 | I rely on my fullness (satiety) signals to tell me when to stop eating | 1.17*** | 0.81 | 0.66 |
|  | IES_23 | I trust my body to tell me when to stop eating | 1.41*** | 0.93 | 0.86 |
| BFCC | IES_18 | Most of the time, I desire to eat nutritious foods | 1.00 | 0.74 | 0.55 |
|  | IES_19 | I mostly eat foods that make my body perform efficiently (well) | 1.33*** | 1.00 | 1.00 |
|  | IES_20 | I mostly eat foods that give my body energy and stamina | 1.10*** | 0.88 | 0.77 |
| *** Significant at p <.001. *B* = Unstandardised factor loadings, *β =* Standardised factor loadings.  UPE = Unconditional permission to eat; EPR: Eating for physical rather than emotional reasons; RHSC = Reliance on hunger & satiety cues; BFCC = Body-food choice congruence | | | | | |

| **S3 Table I** | | | | | |
| --- | --- | --- | --- | --- | --- |
| CFA factor loadings for Model 3: Kidney transplant recipient sample | | | | | |
| Factor | Indicator | Item | *B* | *β* | *R^2^* |
| Global | IES_11 | I find myself eating when I am stressed out, even when I’m not physically hungry | 1.00 | 0.82 | 0.68 |
|  | IES_01 | I try to avoid certain foods high in fat, carbohydrates, or calories | -0.20*** | -0.23 | 0.05 |
|  | IES_02 | I find myself eating when I’m feeling emotional, even when I’m not physically hungry | 0.91*** | 0.76 | 0.57 |
|  | IES_03 | If I am craving a certain food, I allow myself to have it | -0.21*** | -0.22 | 0.05 |
|  | IES_04 | I get mad at myself for eating something unhealthy | 0.67*** | 0.57 | 0.33 |
|  | IES_05 | I find myself eating when I am lonely, even when I’m not physically hungry | 0.89*** | 0.78 | 0.61 |
|  | IES_06 | I trust my body to tell me when to eat | 0.37*** | 0.36 | 0.13 |
|  | IES_07 | I trust my body to tell me what to eat | 0.32*** | 0.33 | 0.11 |
|  | IES_08 | I trust my body to tell me how much to eat | 0.47*** | 0.45 | 0.20 |
|  | IES_09 | I have forbidden foods that I don’t allow myself to eat | -0.01 | -0.01 | 0.00 |
|  | IES_10 | I use food to help me soothe my negative emotions | 0.91*** | 0.82 | 0.67 |
|  | IES_12 | I am able to cope with my negative emotions (e.g. anxiety, sadness) without turning to food for comfort | 0.66*** | 0.62 | 0.39 |
|  | IES_13 | When I am bored, I do not eat just for something to do | 0.61*** | 0.57 | 0.33 |
|  | IES_14 | When I am lonely, I do not turn to food for comfort | 0.75*** | 0.67 | 0.45 |
|  | IES_15 | I find other ways to cope with stress and anxiety than by eating | 0.67*** | 0.67 | 0.44 |
|  | IES_16 | I allow myself to eat what food I desire at the moment | 0.01 | 0.01 | 0.00 |
|  | IES_17 | I do not follow eating rules or dieting plans that dictate what, when and/or how much to eat | 0.11 | 0.10 | 0.01 |
|  | IES_18 | Most of the time, I desire to eat nutritious foods | 0.44*** | 0.47 | 0.22 |
|  | IES_19 | I mostly eat foods that make my body perform efficiently (well) | 0.48*** | 0.53 | 0.28 |
|  | IES_20 | I mostly eat foods that give my body energy and stamina | 0.42*** | 0.48 | 0.23 |
|  | IES_21 | I rely on my hunger signals to tell me when to eat | 0.38*** | 0.38 | 0.14 |
|  | IES_22 | I rely on my fullness (satiety) signals to tell me when to stop eating | 0.46*** | 0.44 | 0.20 |
|  | IES_23 | I trust my body to tell me when to stop eating | 0.53*** | 0.49 | 0.24 |
| *** Significant at p <.001. *B* = Unstandardised factor loadings, *β =* Standardised factor loadings. | | | | | |

| **S3 Table J** | | | | | |
| --- | --- | --- | --- | --- | --- |
| CFA factor loadings for Model 3: Comparison group | | | | | |
| Factor | Indicator | Item | *B* | *β* | *R^2^* |
| Global | IES_11 | I find myself eating when I am stressed out, even when I’m not physically hungry | 1.00 | 0.82 | 0.67 |
|  | IES_01 | I try to avoid certain foods high in fat, carbohydrates, or calories | -0.09 | -0.09 | 0.01 |
|  | IES_02 | I find myself eating when I’m feeling emotional, even when I’m not physically hungry | 0.91*** | 0.77 | 0.59 |
|  | IES_03 | If I am craving a certain food, I allow myself to have it | -0.14 | -0.15 | 0.02 |
|  | IES_04 | I get mad at myself for eating something unhealthy | 0.60*** | 0.54 | 0.29 |
|  | IES_05 | I find myself eating when I am lonely, even when I’m not physically hungry | 0.84*** | 0.73 | 0.54 |
|  | IES_06 | I trust my body to tell me when to eat | 0.58*** | 0.53 | 0.28 |
|  | IES_07 | I trust my body to tell me what to eat | 0.38*** | 0.35 | 0.13 |
|  | IES_08 | I trust my body to tell me how much to eat | 0.60*** | 0.53 | 0.28 |
|  | IES_09 | I have forbidden foods that I don’t allow myself to eat | 0.02 | 0.02 | 0.00 |
|  | IES_10 | I use food to help me soothe my negative emotions | 0.89*** | 0.80 | 0.63 |
|  | IES_12 | I am able to cope with my negative emotions (e.g. anxiety, sadness) without turning to food for comfort | 0.68*** | 0.69 | 0.47 |
|  | IES_13 | When I am bored, I do not eat just for something to do | 0.69*** | 0.59 | 0.35 |
|  | IES_14 | When I am lonely, I do not turn to food for comfort | 0.79*** | 0.69 | 0.48 |
|  | IES_15 | I find other ways to cope with stress and anxiety than by eating | 0.66*** | 0.71 | 0.50 |
|  | IES_16 | I allow myself to eat what food I desire at the moment | 0.00 | 0.00 | 0.00 |
|  | IES_17 | I do not follow eating rules or dieting plans that dictate what, when and/or how much to eat | 0.25*** | 0.20 | 0.04 |
|  | IES_18 | Most of the time, I desire to eat nutritious foods | 0.38*** | 0.36 | 0.13 |
|  | IES_19 | I mostly eat foods that make my body perform efficiently (well) | 0.47*** | 0.45 | 0.20 |
|  | IES_20 | I mostly eat foods that give my body energy and stamina | 0.39*** | 0.40 | 0.16 |
|  | IES_21 | I rely on my hunger signals to tell me when to eat | 0.46*** | 0.46 | 0.21 |
|  | IES_22 | I rely on my fullness (satiety) signals to tell me when to stop eating | 0.47*** | 0.45 | 0.20 |
|  | IES_23 | I trust my body to tell me when to stop eating | 0.58*** | 0.52 | 0.27 |
| *** Significant at p <.001. *B* = Unstandardised factor loadings, *β =* Standardised factor loadings. | | | | | |

| **S3 Table K** | | | | | |
| --- | --- | --- | --- | --- | --- |
| CFA factor loadings for Model 3-CE: Kidney transplant recipient sample | | | | | |
| Factor | Indicator | Item | *B* | *β* | *R^2^* |
| Global | IES_11 | I find myself eating when I am stressed out, even when I’m not physically hungry | 1.00 | 0.74 | 0.55 |
|  | IES_01 | I try to avoid certain foods high in fat, carbohydrates, or calories | -0.25*** | -0.25 | 0.06 |
|  | IES_02 | I find myself eating when I’m feeling emotional, even when I’m not physically hungry | 0.88*** | 0.66 | 0.43 |
|  | IES_03 | If I am craving a certain food, I allow myself to have it | -0.23*** | -0.21 | 0.04 |
|  | IES_04 | I get mad at myself for eating something unhealthy | 0.71*** | 0.55 | 0.30 |
|  | IES_05 | I find myself eating when I am lonely, even when I’m not physically hungry | 0.91*** | 0.72 | 0.51 |
|  | IES_06 | I trust my body to tell me when to eat | 0.38*** | 0.33 | 0.11 |
|  | IES_07 | I trust my body to tell me what to eat | 0.34*** | 0.31 | 0.09 |
|  | IES_08 | I trust my body to tell me how much to eat | 0.49*** | 0.43 | 0.18 |
|  | IES_09 | I have forbidden foods that I don’t allow myself to eat | -0.03 | -0.02 | 0.00 |
|  | IES_10 | I use food to help me soothe my negative emotions | 0.96*** | 0.78 | 0.60 |
|  | IES_12 | I am able to cope with my negative emotions (e.g. anxiety, sadness) without turning to food for comfort | 0.76*** | 0.64 | 0.41 |
|  | IES_13 | When I am bored, I do not eat just for something to do | 0.74*** | 0.63 | 0.39 |
|  | IES_14 | When I am lonely, I do not turn to food for comfort | 0.91*** | 0.73 | 0.53 |
|  | IES_15 | I find other ways to cope with stress and anxiety than by eating | 0.81*** | 0.72 | 0.52 |
|  | IES_16 | I allow myself to eat what food I desire at the moment | 0.03 | 0.03 | 0.00 |
|  | IES_17 | I do not follow eating rules or dieting plans that dictate what, when and/or how much to eat | 0.14 | 0.12 | 0.01 |
|  | IES_18 | Most of the time, I desire to eat nutritious foods | 0.54*** | 0.52 | 0.27 |
|  | IES_19 | I mostly eat foods that make my body perform efficiently (well) | 0.58*** | 0.57 | 0.32 |
|  | IES_20 | I mostly eat foods that give my body energy and stamina | 0.48*** | 0.50 | 0.25 |
|  | IES_21 | I rely on my hunger signals to tell me when to eat | 0.43*** | 0.38 | 0.15 |
|  | IES_22 | I rely on my fullness (satiety) signals to tell me when to stop eating | 0.52*** | 0.45 | 0.20 |
|  | IES_23 | I trust my body to tell me when to stop eating | 0.59*** | 0.49 | 0.24 |
| *** Significant at p <.001. *B* = Unstandardised factor loadings, *β =* Standardised factor loadings. | | | | | |

| **S3 Table L** | | | | | |
| --- | --- | --- | --- | --- | --- |
| CFA factor loadings for Model 3-CE: Comparison group | | | | | |
| Factor | Indicator | Item | *B* | *β* | *R^2^* |
| Global | IES_11 | I find myself eating when I am stressed out, even when I’m not physically hungry | 1.00 | 0.79 | 0.63 |
|  | IES_01 | I try to avoid certain foods high in fat, carbohydrates, or calories | -0.09 | -0.09 | 0.01 |
|  | IES_02 | I find myself eating when I’m feeling emotional, even when I’m not physically hungry | 0.89*** | 0.73 | 0.53 |
|  | IES_03 | If I am craving a certain food, I allow myself to have it | -0.15* | -0.16 | 0.03 |
|  | IES_04 | I get mad at myself for eating something unhealthy | 0.61*** | 0.53 | 0.28 |
|  | IES_05 | I find myself eating when I am lonely, even when I’m not physically hungry | 0.84*** | 0.71 | 0.50 |
|  | IES_06 | I trust my body to tell me when to eat | 0.56*** | 0.49 | 0.24 |
|  | IES_07 | I trust my body to tell me what to eat | 0.33*** | 0.30 | 0.09 |
|  | IES_08 | I trust my body to tell me how much to eat | 0.58*** | 0.49 | 0.24 |
|  | IES_09 | I have forbidden foods that I don’t allow myself to eat | 0.03 | 0.02 | 0.00 |
|  | IES_10 | I use food to help me soothe my negative emotions | 0.93*** | 0.80 | 0.64 |
|  | IES_12 | I am able to cope with my negative emotions (e.g. anxiety, sadness) without turning to food for comfort | 0.72*** | 0.71 | 0.50 |
|  | IES_13 | When I am bored, I do not eat just for something to do | 0.75*** | 0.61 | 0.38 |
|  | IES_14 | When I am lonely, I do not turn to food for comfort | 0.85*** | 0.71 | 0.51 |
|  | IES_15 | I find other ways to cope with stress and anxiety than by eating | 0.70*** | 0.73 | 0.53 |
|  | IES_16 | I allow myself to eat what food I desire at the moment | -0.01 | -0.01 | 0.00 |
|  | IES_17 | I do not follow eating rules or dieting plans that dictate what, when and/or how much to eat | 0.25*** | 0.20 | 0.04 |
|  | IES_18 | Most of the time, I desire to eat nutritious foods | 0.37*** | 0.34 | 0.11 |
|  | IES_19 | I mostly eat foods that make my body perform efficiently (well) | 0.46*** | 0.42 | 0.18 |
|  | IES_20 | I mostly eat foods that give my body energy and stamina | 0.38*** | 0.37 | 0.14 |
|  | IES_21 | I rely on my hunger signals to tell me when to eat | 0.44*** | 0.43 | 0.18 |
|  | IES_22 | I rely on my fullness (satiety) signals to tell me when to stop eating | 0.48*** | 0.44 | 0.19 |
|  | IES_23 | I trust my body to tell me when to stop eating | 0.58*** | 0.50 | 0.25 |
| *** Significant at p <.001. * Significant at p <.05. *B* = Unstandardised factor loadings, *β =* Standardised factor loadings. | | | | | |

| **S3 Table M** | | | | | |
| --- | --- | --- | --- | --- | --- |
| CFA factor loadings for Model 4: Kidney transplant recipient sample | | | | | |
| Factor | Indicator | Item | *B* | *β* | *R^2^* |
| AFF | IES_01 | I try to avoid certain foods high in fat, carbohydrates, or calories | 1.00 | 0.07 | 0.00 |
|  | IES_09 | I have forbidden foods that I don’t allow myself to eat | 0.12 | 0.01 | 0.00 |
|  | IES_04 | I get mad at myself for eating something unhealthy | -2.15*** | -0.11 | 0.01 |
| PTE | IES_03 | If I am craving a certain food, I allow myself to have it | 1.00 | 0.58 | 0.34 |
|  | IES_16 | I allow myself to eat what food I desire at the moment | 1.37*** | 0.83 | 0.69 |
|  | IES_17 | I do not follow eating rules or dieting plans that dictate what, when and/or how much to eat | 0.78*** | 0.41 | 0.17 |
| AEE | IES_02 | I find myself eating when I’m feeling emotional, even when I’m not physically hungry | 1.00 | 0.83 | 0.69 |
|  | IES_05 | I find myself eating when I am lonely, even when I’m not physically hungry | 0.91*** | 0.81 | 0.65 |
|  | IES_10 | I use food to help me soothe my negative emotions | 0.96*** | 0.87 | 0.75 |
|  | IES_11 | I find myself eating when I am stressed out, even when I’m not physically hungry | 1.10*** | 0.91 | 0.83 |
| AFRCS | IES_12 | I am able to cope with my negative emotions (e.g. anxiety, sadness) without turning to food for comfort | 1.00 | 0.64 | 0.41 |
|  | IES_13 | When I am bored, I do not eat just for something to do | 1.08*** | 0.69 | 0.48 |
|  | IES_14 | When I am lonely, I do not turn to food for comfort | 1.30*** | 0.79 | 0.62 |
|  | IES_15 | I find other ways to cope with stress and anxiety than by eating | 1.13*** | 0.76 | 0.58 |
| RHSC | IES_06 | I trust my body to tell me when to eat | 1.00 | 0.61 | 0.38 |
|  | IES_07 | I trust my body to tell me what to eat | 0.94*** | 0.60 | 0.36 |
|  | IES_08 | I trust my body to tell me how much to eat | 1.25*** | 0.76 | 0.58 |
|  | IES_21 | I rely on my hunger signals to tell me when to eat | 1.03*** | 0.64 | 0.41 |
|  | IES_22 | I rely on my fullness (satiety) signals to tell me when to stop eating | 1.20*** | 0.72 | 0.52 |
|  | IES_23 | I trust my body to tell me when to stop eating | 1.38*** | 0.80 | 0.64 |
| BFCC | IES_18 | Most of the time, I desire to eat nutritious foods | 1.00*** | 0.77 | 0.60 |
|  | IES_19 | I mostly eat foods that make my body perform efficiently (well) | 1.20*** | 0.96 | 0.91 |
|  | IES_20 | I mostly eat foods that give my body energy and stamina | 0.93*** | 0.77 | 0.60 |
| *** Significant at p <.001. *B* = Unstandardised factor loadings, *β =* Standardised factor loadings.  AFF = Avoiding forbidden foods; PTE = Permission to eat; AEE = Avoiding emotional eating; AFRCS = Avoiding food-related coping strategies;  RHSC = Reliance on hunger & satiety cues; BFCC = Body-food choice congruence | | | | | |

| **S3 Table N** | | | | | |
| --- | --- | --- | --- | --- | --- |
| CFA factor loadings for Model 4: Comparison group | | | | | |
| Factor | Indicator | Item | *B* | *β* | *R^2^* |
| AFF | IES_01 | I try to avoid certain foods high in fat, carbohydrates, or calories | 1.00 | 0.53 | 0.28 |
|  | IES_09 | I have forbidden foods that I don’t allow myself to eat | 1.26*** | 0.60 | 0.36 |
|  | IES_04 | I get mad at myself for eating something unhealthy | 0.87*** | 0.44 | 0.20 |
| PTE | IES_03 | If I am craving a certain food, I allow myself to have it | 1.00 | 0.72 | 0.52 |
|  | IES_16 | I allow myself to eat what food I desire at the moment | 1.04*** | 0.73 | 0.54 |
|  | IES_17 | I do not follow eating rules or dieting plans that dictate what, when and/or how much to eat | 1.08*** | 0.55 | 0.30 |
| AEE | IES_02 | I find myself eating when I’m feeling emotional, even when I’m not physically hungry | 1.00 | 0.85 | 0.72 |
|  | IES_05 | I find myself eating when I am lonely, even when I’m not physically hungry | 0.87*** | 0.76 | 0.58 |
|  | IES_10 | I use food to help me soothe my negative emotions | 0.96*** | 0.85 | 0.73 |
|  | IES_11 | I find myself eating when I am stressed out, even when I’m not physically hungry | 1.12*** | 0.92 | 0.84 |
| AFRCS | IES_12 | I am able to cope with my negative emotions (e.g. anxiety, sadness) without turning to food for comfort | 1.00 | 0.76 | 0.58 |
|  | IES_13 | When I am bored, I do not eat just for something to do | 1.01*** | 0.65 | 0.42 |
|  | IES_14 | When I am lonely, I do not turn to food for comfort | 1.16*** | 0.75 | 0.57 |
|  | IES_15 | I find other ways to cope with stress and anxiety than by eating | 0.94*** | 0.76 | 0.58 |
| RHSC | IES_06 | I trust my body to tell me when to eat | 1.00 | 0.74 | 0.55 |
|  | IES_07 | I trust my body to tell me what to eat | 0.76*** | 0.58 | 0.33 |
|  | IES_08 | I trust my body to tell me how much to eat | 1.14*** | 0.82 | 0.67 |
|  | IES_21 | I rely on my hunger signals to tell me when to eat | 0.85*** | 0.69 | 0.48 |
|  | IES_22 | I rely on my fullness (satiety) signals to tell me when to stop eating | 0.97*** | 0.75 | 0.57 |
|  | IES_23 | I trust my body to tell me when to stop eating | 1.12*** | 0.82 | 0.68 |
| BFCC | IES_18 | Most of the time, I desire to eat nutritious foods | 1.00 | 0.75 | 0.57 |
|  | IES_19 | I mostly eat foods that make my body perform efficiently (well) | 1.28*** | 0.98 | 0.96 |
|  | IES_20 | I mostly eat foods that give my body energy and stamina | 1.06*** | 0.87 | 0.75 |
| *** Significant at p <.001. *B* = Unstandardised factor loadings, *β =* Standardised factor loadings.  AFF = Avoiding forbidden foods; PTE = Permission to eat; AEE = Avoiding emotional eating; AFRCS = Avoiding food-related coping strategies;  RHSC = Reliance on hunger & satiety cues; BFCC = Body-food choice congruence | | | | | |

| **S3 Table O** | | | | | |
| --- | --- | --- | --- | --- | --- |
| CFA factor loadings for Model 4-CE: Kidney transplant recipient sample | | | | | |
| Factor | Indicator | Item | *B* | *β* | *R^2^* |
| AFF | IES_01 | I try to avoid certain foods high in fat, carbohydrates, or calories | 1.00 | 0.07 | 0.004 |
|  | IES_09 | I have forbidden foods that I don’t allow myself to eat | 0.09 | 0.004 | 0.00 |
|  | IES_04 | I get mad at myself for eating something unhealthy | -2.01*** | -0.10 | 0.01 |
| PTE | IES_03 | If I am craving a certain food, I allow myself to have it | 1.00 | 0.57 | 0.33 |
|  | IES_16 | I allow myself to eat what food I desire at the moment | 1.41*** | 0.84 | 0.70 |
|  | IES_17 | I do not follow eating rules or dieting plans that dictate what, when and/or how much to eat | 0.80*** | 0.42 | 0.18 |
| AEE | IES_02 | I find myself eating when I’m feeling emotional, even when I’m not physically hungry | 1.00 | 0.79 | 0.63 |
|  | IES_05 | I find myself eating when I am lonely, even when I’m not physically hungry | 0.97*** | 0.81 | 0.66 |
|  | IES_10 | I use food to help me soothe my negative emotions | 1.03*** | 0.88 | 0.78 |
|  | IES_11 | I find myself eating when I am stressed out, even when I’m not physically hungry | 1.12*** | 0.89 | 0.79 |
| AFRCS | IES_12 | I am able to cope with my negative emotions (e.g. anxiety, sadness) without turning to food for comfort | 1.00 | 0.67 | 0.45 |
|  | IES_13 | When I am bored, I do not eat just for something to do | 1.03*** | 0.69 | 0.48 |
|  | IES_14 | When I am lonely, I do not turn to food for comfort | 1.28*** | 0.81 | 0.66 |
|  | IES_15 | I find other ways to cope with stress and anxiety than by eating | 1.06*** | 0.75 | 0.56 |
| RHSC | IES_06 | I trust my body to tell me when to eat | 1.00 | 0.53 | 0.28 |
|  | IES_07 | I trust my body to tell me what to eat | 0.91*** | 0.50 | 0.25 |
|  | IES_08 | I trust my body to tell me how much to eat | 1.30*** | 0.69 | 0.47 |
|  | IES_21 | I rely on my hunger signals to tell me when to eat | 1.17*** | 0.63 | 0.40 |
|  | IES_22 | I rely on my fullness (satiety) signals to tell me when to stop eating | 1.46*** | 0.76 | 0.57 |
|  | IES_23 | I trust my body to tell me when to stop eating | 1.77*** | 0.89 | 0.79 |
| BFCC | IES_18 | Most of the time, I desire to eat nutritious foods | 1.00 | 0.83 | 0.69 |
|  | IES_19 | I mostly eat foods that make my body perform efficiently (well) | 1.05*** | 0.90 | 0.80 |
|  | IES_20 | I mostly eat foods that give my body energy and stamina | 0.78*** | 0.70 | 0.48 |
| *** Significant at p <.001. *B* = Unstandardised factor loadings, *β =* Standardised factor loadings.  AFF = Avoiding forbidden foods; PTE = Permission to eat; AEE = Avoiding emotional eating; AFRCS = Avoiding food-related coping strategies;  RHSC = Reliance on hunger & satiety cues; BFCC = Body-food choice congruence | | | | | |

| **S3 Table P** | | | | | |
| --- | --- | --- | --- | --- | --- |
| CFA factor loadings for Model 4-CE: Comparison group | | | | | |
| Factor | Indicator | Item | *B* | *β* | *R^2^* |
| AFF | IES_01 | I try to avoid certain foods high in fat, carbohydrates, or calories | 1.00 | 0.54 | 0.77 |
|  | IES_09 | I have forbidden foods that I don’t allow myself to eat | 1.27*** | 0.61 | 1.00 |
|  | IES_04 | I get mad at myself for eating something unhealthy | 0.82*** | 0.43 | 0.55 |
| PTE | IES_03 | If I am craving a certain food, I allow myself to have it | 1.00 | 0.72 | 0.86 |
|  | IES_16 | I allow myself to eat what food I desire at the moment | 1.04*** | 0.74 | 0.66 |
|  | IES_17 | I do not follow eating rules or dieting plans that dictate what, when and/or how much to eat | 1.07*** | 0.54 | 0.42 |
| AEE | IES_02 | I find myself eating when I’m feeling emotional, even when I’m not physically hungry | 1.00 | 0.81 | 0.57 |
|  | IES_05 | I find myself eating when I am lonely, even when I’m not physically hungry | 0.90*** | 0.74 | 0.21 |
|  | IES_10 | I use food to help me soothe my negative emotions | 1.04*** | 0.88 | 0.44 |
|  | IES_11 | I find myself eating when I am stressed out, even when I’m not physically hungry | 1.13*** | 0.88 | 0.57 |
| AFRCS | IES_12 | I am able to cope with my negative emotions (e.g. anxiety, sadness) without turning to food for comfort | 1.00 | 0.77 | 0.59 |
|  | IES_13 | When I am bored, I do not eat just for something to do | 1.01*** | 0.65 | 0.42 |
|  | IES_14 | When I am lonely, I do not turn to food for comfort | 1.16*** | 0.77 | 0.59 |
|  | IES_15 | I find other ways to cope with stress and anxiety than by eating | 0.92*** | 0.75 | 0.78 |
| RHSC | IES_06 | I trust my body to tell me when to eat | 1.00 | 0.66 | 0.77 |
|  | IES_07 | I trust my body to tell me what to eat | 0.68*** | 0.46 | 0.55 |
|  | IES_08 | I trust my body to tell me how much to eat | 1.18*** | 0.75 | 0.65 |
|  | IES_21 | I rely on my hunger signals to tell me when to eat | 0.89*** | 0.65 | 0.30 |
|  | IES_22 | I rely on my fullness (satiety) signals to tell me when to stop eating | 1.18*** | 0.81 | 0.54 |
|  | IES_23 | I trust my body to tell me when to stop eating | 1.42*** | 0.93 | 0.38 |
| BFCC | IES_18 | Most of the time, I desire to eat nutritious foods | 1.00 | 0.74 | 0.18 |
|  | IES_19 | I mostly eat foods that make my body perform efficiently (well) | 1.33*** | 1.00 | 0.52 |
|  | IES_20 | I mostly eat foods that give my body energy and stamina | 1.10*** | 0.88 | 0.29 |
| *** Significant at p <.001. *B* = Unstandardised factor loadings, *β =* Standardised factor loadings.  AFF = Avoiding forbidden foods; PTE = Permission to eat; AEE = Avoiding emotional eating; AFRCS = Avoiding food-related coping strategies;  RHSC = Reliance on hunger & satiety cues; BFCC = Body-food choice congruence | | | | | |

| **S3 Table Q** | | | | | |
| --- | --- | --- | --- | --- | --- |
| CFA factor loadings for Model 5: Kidney transplant recipient sample | | | | | |
| Factor | Indicator | Item | *B* | *β* | *R^2^* |
| EPR | IES_02 | I find myself eating when I’m feeling emotional, even when I’m not physically hungry | 1.00 | 0.84 | 0.59 |
|  | IES_05 | I find myself eating when I am lonely, even when I’m not physically hungry | 0.90*** | 0.80 | 0.95 |
|  | IES_10 | I use food to help me soothe my negative emotions | 0.95*** | 0.86 | 0.58 |
|  | IES_11 | I find myself eating when I am stressed out, even when I’m not physically hungry | 1.10*** | 0.92 | 0.42 |
| RHSC | IES_06 | I trust my body to tell me when to eat | 1.00 | 0.68 | 0.72 |
|  | IES_07 | I trust my body to tell me what to eat | 0.99*** | 0.70 | 0.49 |
|  | IES_08 | I trust my body to tell me how much to eat | 1.26*** | 0.85 | 0.46 |
|  | IES_23 | I trust my body to tell me when to stop eating | 1.00*** | 0.65 | 0.85 |
| BFCC | IES_18 | Most of the time, I desire to eat nutritious foods | 1.00 | 0.76 | 0.74 |
|  | IES_19 | I mostly eat foods that make my body perform efficiently (well) | 1.24*** | 0.97 | 0.64 |
|  | IES_20 | I mostly eat foods that give my body energy and stamina | 0.93*** | 0.77 | 0.70 |
| *** Significant at p <.001. *B* = Unstandardised factor loadings, *β =* Standardised factor loadings.  EPR = Eating for physical rather than emotional reasons; RHSC = Reliance on hunger & satiety cues; BFCC = Body-food choice congruence | | | | | |

| **S3 Table R** | | | | | |
| --- | --- | --- | --- | --- | --- |
| CFA factor loadings for Model 5: Comparison group | | | | | |
| Factor | Indicator | Item | *B* | *β* | *R^2^* |
| EPR | IES_02 | I find myself eating when I’m feeling emotional, even when I’m not physically hungry | 1.00 | 0.85 | 0.76 |
|  | IES_05 | I find myself eating when I am lonely, even when I’m not physically hungry | 0.86*** | 0.75 | 0.95 |
|  | IES_10 | I use food to help me soothe my negative emotions | 0.95*** | 0.84 | 0.57 |
|  | IES_11 | I find myself eating when I am stressed out, even when I’m not physically hungry | 1.13*** | 0.93 | 0.49 |
| RHSC | IES_06 | I trust my body to tell me when to eat | 1.00 | 0.79 | 0.75 |
|  | IES_07 | I trust my body to tell me what to eat | 0.81*** | 0.66 | 0.44 |
|  | IES_08 | I trust my body to tell me how much to eat | 1.13*** | 0.87 | 0.63 |
|  | IES_23 | I trust my body to tell me when to stop eating | 0.89*** | 0.70 | 0.86 |
| BFCC | IES_18 | Most of the time, I desire to eat nutritious foods | 1.00 | 0.76 | 0.71 |
|  | IES_19 | I mostly eat foods that make my body perform efficiently (well) | 1.26*** | 0.97 | 0.56 |
|  | IES_20 | I mostly eat foods that give my body energy and stamina | 1.06*** | 0.87 | 0.72 |
| *** Significant at p <.001. *B* = Unstandardised factor loadings, *β =* Standardised factor loadings.  EPR = Eating for physical rather than emotional reasons; RHSC = Reliance on hunger & satiety cues; BFCC = Body-food choice congruence | | | | | |
